# Genomic Insights Into Respiratory Syncytial Virus Circulation Patterns and Neutralization by Anti‐F Monoclonal Antibodies in Panama (2018–2024)

**DOI:** 10.1111/irv.70173

**Published:** 2025-10-22

**Authors:** Danilo Franco, Stephanie Goya, Alexander Martínez, Vicente Mas, Brechla Moreno, Elimelec Valdespino, Melissa Gaitán, Lisseth Sáenz, Claudia González, Ambar Moreno, Zeuz Capitan‐Barrios, Jean Paul Carrera, Sandra López‐Vergès, Juan Miguel Pascale, Yadira Moltó, Lourdes Moreno, Belmaris Rizo, Enrique Urriola, Teresa Delgado, María Iglesias‐Caballero, Inmaculada Casas, Suman R. Das, Juan Arbiza, Adriana Delfraro, Leyda Ábrego

**Affiliations:** ^1^ Modular Specialized Laboratory, Department of Research in Virology and Biotechnology Gorgas Memorial Institute for Health Studies Panama City Panama; ^2^ Programa de Desarrollo en Ciencias Básicas (PEDECIBA), Universidad de La República Montevideo Uruguay; ^3^ Department of Laboratory Medicine and Pathology University of Washington Seattle Washington USA; ^4^ Department of Genomic and Proteomic Gorgas Memorial Institute of Health Studies Panama City Panama; ^5^ National Research System (SNI), National Secretary of Research, Technology and Innovation (SENACYT) Panama City Panama; ^6^ Departamento de Microbiología, Facultad de Medicina, Escuela de Medicina Universidad de Panamá Panama City Panama; ^7^ Laboratory of Reference and Research in Respiratory Viruses, National Centre for Microbiology Instituto de Salud Carlos III Majadahonda Spain; ^8^ Virology Research Laboratory, Department of Research in Virology and Biotechnology Gorgas Memorial Institute for Health Studies Panama City Panama; ^9^ Facultad de Ciencias Naturales, Exactas y Tecnología, Departamento de Microbiología y Parasitología Universidad de Panamá Panama City Panamá; ^10^ Carson Centre for Research in Environment and Emerging Infectious Diseases Darien Panama; ^11^ Centro Regional de Innovación en Vacunas y Biofármacos, CRIVB AIP Panama City Panama; ^12^ National Department of Epidemiology Ministry of Health Panama Panama; ^13^ Escuela de Tecnología Médica, Facultad de Medicina Universidad de Panamá Panama City Panama; ^14^ Department of Medicine Vanderbilt University Medical Center Nashville Tennessee USA; ^15^ Sección Virología, Facultad de Ciencias Universidad de La República Montevideo Uruguay

**Keywords:** lineages, Nirsevimab, respiratory syncytial virus

## Abstract

**Background:**

Respiratory syncytial virus (RSV) is a leading cause of lower respiratory tract infections and hospitalization in infants and children. Whole genome sequencing (WGS) plays a critical role in understanding the evolution and epidemiology of RSV. Limited studies have been conducted in Central America and the Caribbean, and none have specifically focused on lineages involved in recent outbreaks. Furthermore, no assays currently exist to evaluate the sensitivity of the RSV fusion protein to monoclonal antibodies.

**Methods:**

In Panama, an epidemiological surveillance system tracks RSV activity through the collection of nasopharyngeal samples from patients with acute respiratory infections. Between January 2018 and July 2024, 303 RSV‐positive samples were analyzed by RT‐qPCR. RSV‐B was the dominant subgroup in 2018, but following years had alternating dominance between RSV‐A and RSV‐B. Of the 303 samples, 115 underwent WGS. Additionally, neutralization assays were done using different Anti‐F Monoclonal Antibodies.

**Results:**

In RSV‐A, 11 lineages were identified, with 3 to 5 cocirculating during each annual outbreak, and a shift in predominance from a.d.1 (2019) to a.d.5.2 (2023–2024). In RSV‐B, two lineages circulated: B.D.4.1.1 (2018–2020) and its descendant B.D.E.1, which predominated from 2021 onward. Several monoclonal antibodies, including nirsevimab's precursor MEDI8897*, effectively neutralized the RSV strains in neutralization assays.

**Conclusions:**

Although Panama has not yet implemented a preventive therapy for RSV, this step could modify outbreak dynamics. The findings from this study provide a baseline reference prior to the implementation of preventive therapies against RSV in Panama and the region, facilitating the assessment of potential changes in the evolutionary dynamics of the virus.

## 1 Introduction

Human respiratory syncytial virus (RSV) is the leading cause of severe acute respiratory infections (SARIs) in children worldwide. In Panama, RSV accounts for 21% of acute respiratory infections in children under 2 years of age, the highest incidence among respiratory viruses, with 10% of these cases requiring hospitalization [1].

RSV belongs to the Pneumoviridae family, *Orthopneumovirus* genu*s*. It is an enveloped virus with a nonsegmented, negative‐sense, single‐stranded RNA genome of approximately 15.2 kb [2]. The genome consists of 10 genes encoding 11 proteins: nine structural and two nonstructural proteins. RSV has two antigenic subgroups, RSV‐A and RSV‐B, which were initially subdivided into several genotypes based on the attachment glycoprotein (G) gene [3]. Previous RSV surveillance in Panama (2008–2012), based on the G attachment glycoprotein sequence, identified the predominance of the GA2 genotype and the emergence of the ON1 variant (characterized by a 72‐nucleotide insertion) in the RSV‐A subgroup. Despite the observed genetic variability, the G protein remained antigenically stable [4]. In the RSV‐B subgroup, this surveillance reported the emergence of the BA14 genotype [5]. More recently, with the advancement of next‐generation sequencing platforms and bioinformatics analyses, and with the aim of standardizing classification and strengthening molecular surveillance, new classification systems for RSV have been proposed. These systems are based on phylogenetic associations derived from whole genome analysis [6, 7] and also incorporate information on amino acid signatures, proposing a transition from genotypes to lineages [8]. In Panama, comprehensive genomic data on circulating RSV strains are still lacking, and in fact, as of December 2024, genomic data from Central America and the Caribbean remain limited, representing less than 0.5% of all RSV genome submissions on the GISAID platform [9]. This scarcity of data hinders our ability to draw robust conclusions about RSV evolutionary dynamics in the region, especially in the context of the COVID‐19 pandemic, which globally reduced RSV transmission and may have influenced viral diversity and selection [10, 11].

With the imminent implementation of preventive treatment against RSV in Panama and in other countries of the region [12, 13, 14], monitoring the genomic evolution will be decisive in the context of public health. Particularly important is tracking the evolution of the fusion F protein, the primary target for monoclonal antibodies (mAb) and vaccines [15]. The F protein contains six antigenic sites (Ø, I–V) that have been widely studied [16, 17], which has enabled the development of neutralizing mAbs as a preventive treatment. Palivizumab was the first mAb authorized for RSV prophylaxis, and it targets antigenic site II [18]. The recently approved nirsevimab targets the Ø site and has a half‐life of 150 days [19, 20]. Another novel mAb is clesrovimab (MK‐1654), currently in the final stages of approval. It binds to the highly conserved antigenic site IV of the RSV F protein, emerging as an innovative tool for immunoprophylaxis [15, 21].

In this study, we present the first comprehensive analysis of RSV genomic evolution in Panama spanning the years 2018 to 2024. In addition, we integrate molecular epidemiology data with neutralization assays against the RSV F protein, demonstrating that the circulating lineages are sensitive to recently approved, preventive monoclonal antibodies. These findings not only advance local understanding but also provide critical insights for Central America, the Caribbean, and the global context, particularly regarding the implementation of RSV immunization strategies.

## 2 Materials and Methods

### 2.1 Ethics Statement

This study was approved by the Ethics Committee of the Gorgas Memorial Institute for Health Studies, Panama City, Panama (219/CBI/ICGES/22 and amendment 300/CBI/ICGES/24).

### 2.2 Epidemiological Surveillance System in Panama

**FIGURE 1:**
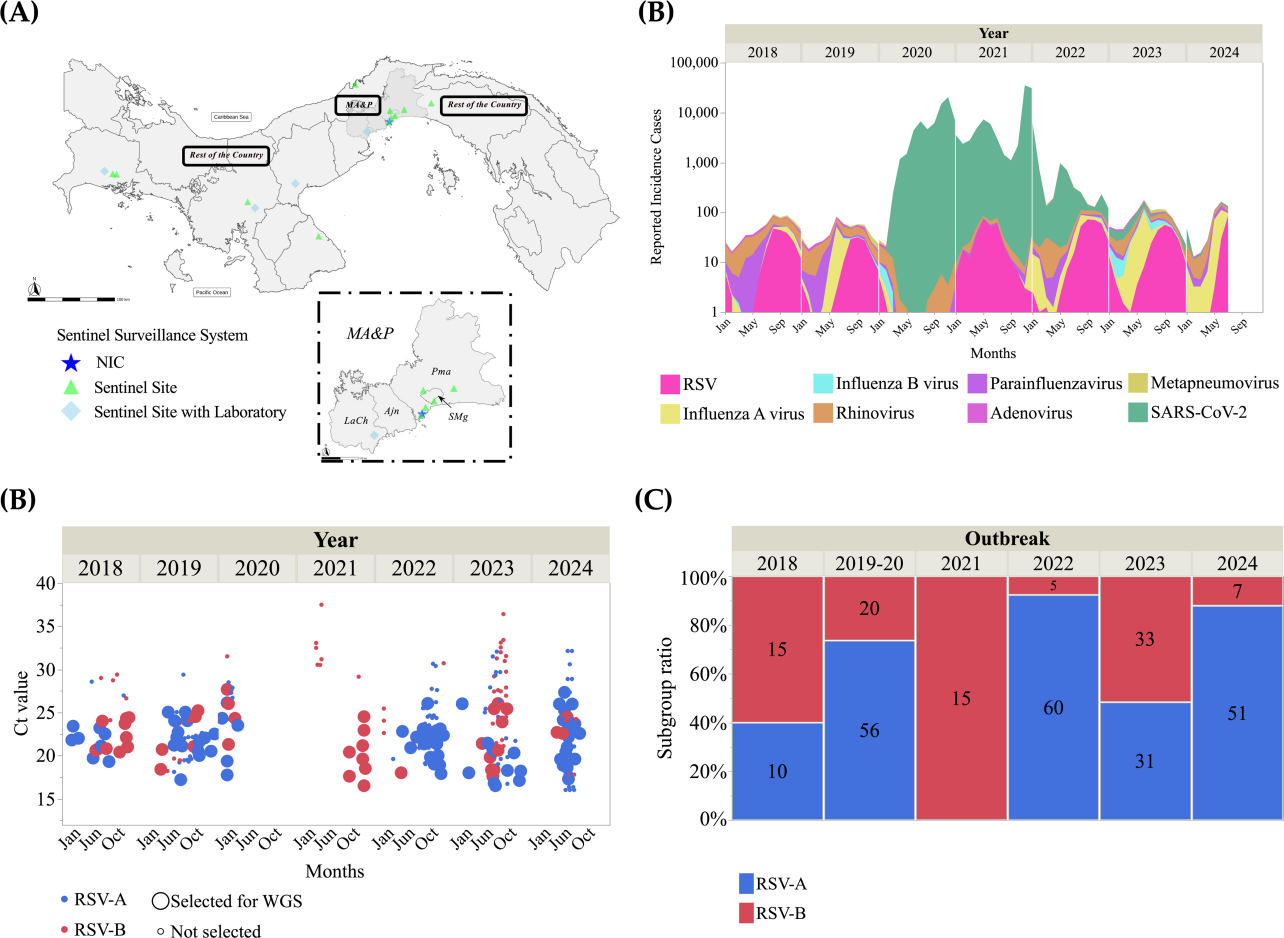
(A) A geographic map of Panama is presented, showing the location of the institutions that collect samples for the National Epidemiological Surveillance System. This system consists of 18 sentinel sites, some of which have sentinel laboratories. Additionally, the location of the National Influenza Center, which is located in the MA&P area, is shown. (B) Respiratory virus surveillance by the National Epidemiological Surveillance System using real‐time RT‐qPCR. During 2020, with the onset of the COVID‐19 pandemic, the system focused on detecting SARS‐CoV‐2, adopting universal surveillance. However, the monitoring of other respiratory viruses in samples from sentinel sites continued. (C) RT‐qPCR Ct values of samples analyzed in this study of each RSV subgroup and selected for WGS. (D) RSV subgroup annual seasonality from 303 RSV‐positive samples using real‐time RT‐PCR. The graphs were plotted in Jump Statistical Software 18.0.1. MA&P indicates metropolitan area and its periphery; Pma, Panama; SMg, San Miguelito; Ajn, Arraijan; LCh, La Chorrera; RSV, respiratory syncytial virus; SARS‐CoV‐2, severe acute respiratory syndrome coronavirus 2; RSV‐A, respiratory syncytial virus subgroup A; RSV‐B, respiratory syncytial virus subgroup B.

Respiratory virus surveillance in Panama, established since 1976 [22], is currently conducted through the National Epidemiological Surveillance System for influenza‐like illness (ILI) syndrome and SARIs. This system managed by the Ministry of Health includes 18 sentinel sites across the country: 8 for ILI and 10 for SARI (Figure 1) [23]. Each week, sentinel sites collect at least five nasopharyngeal swab (NPS) samples from suspected ILI and SARI cases, to be send to the sentinel laboratories or to the National Influenza Center at the Gorgas Memorial Institute in Panama City [23]. This surveillance system seeks to ensure demographic representativity by including samples from all provinces in the country, temporal diversity with sample collection throughout the entire season, and population diversity through the sampling of different age‐groups and health conditions, covering both outpatient and hospitalized cases [23].

### 2.3 Clinical Samples, Viral RNA Extraction, and RT‐qPCR

Between January 2018 and July 2024, a total of 303 RSV clinical samples were retrospectively retrieved from the surveillance system biobank for further subgroup characterization and genome sequencing. The samples include 25 from 2018, 76 from 2019 to 20, 15 from 2021, 65 from 2022, 64 from 2023, and 58 from 2024. Total RNA was extracted using KingFisher Flex (Thermo Fisher Scientific, Rocklin, CA, USA), and RSV subgroup was identified by RT‐qPCR as previously described [24]. From them, a total 115 specimens with Ct < 28 were selected for further analysis, to ensure genome recovery.

### 2.4 Whole Genome Sequencing (WGS)

WGS was performed as previously described [25]. Briefly, cDNA was generated using the SuperScript IV First‐Strand Synthesis System (SSIV, Thermo Fisher Scientific, MA, USA) with four RSV forward primers. Four independent PCR reactions were performed using Phusion Hot Start Flex 2X Master Mix kit (New England Biolabs, Ipswich, MA, USA) to generate four overlapping amplicons of approximately 4 kb length, spanning the complete RSV genome. Amplicons were verified on a 1% agarose gel, pooled, and used for library prep using Nextera XT library kit (Illumina, USA cat. FC‐131‐1096). Libraries were sequenced with a 2 × 250 paired‐end run on an Illumina MiSeq sequencer.

### 2.5 Next‐Generation Sequencing Analysis

We filtered FASTQ files for reads with average quality score above Q30 and minimum length 200 bp using Fastp (S. [26]). Complete genomes were generated by mapping the filtered FASTQ against the references RSV‐A GenBank accession number ON237358.1 or RSV‐B GenBank accession number ON237214.1 using gencomvariable v.2.0 pipeline (https://github.com/AAMCgenomics/gencomvariable.git).

### 2.6 Phylogenetic Analysis

Complete reference genomes of RSV‐A and RSV‐B were downloaded from the RSV Genotyping Consensus Consortium (https://github.com/rsv‐lineages) accessed on August 26, 2024 (supplemental material), and the sequences were compiled according to subgroups and then aligned using MAFFT v7.490 [27] and visualized with Aliview v.1.28 [28]. The best nucleotide substitution model was evaluated with ModelFinder [29], and the maximum likelihood phylogenetic trees were inferred using IQ‐TREE v2.2.0 [30, 31]. Branch support was assessed by 1000 ultrafast bootstrap iterations [31]. Trees were visualized with FigTree v1.4.4 (http://tree.bio.ed.ac.uk/software/figtree).

### 2.7 Viral Isolation

A total of 115 clinical samples selected for RSV whole genome sequencing (Ct < 28) were aliquoted and diluted in Dulbecco's modified Eagle's medium (DMEM) supplemented with 2% inactivated fetal calf serum (FCS) and used to infect HEp‐2 cell monolayers in 12‐well plates as previously described [32]. In the absence of cytopathic effect, cultures were subjected to up to five serial passages. When cytopathic effect was observed, cells were scraped into the medium, and the suspension was stored in liquid nitrogen. The presence of cytopathic effect was confirmed as RSV infection by RT‐qPCR.

### 2.8 Neutralization Assay

Neutralization assays were performed to determine the half‐maximal inhibitory concentration (IC_50_) of three highly potent neutralizing antibodies: MEDI8897* (parental nirsevimab) [20]; motavizumab, a derivative of palivizumab with enhanced neutralizing potency [33, 34]; and AM14 [35], which binds to F protein similarly to Clesrovimab. As an irrelevant control, the anti‐F metapneumovirus antibody MF14 [36] was included. The antibodies were tested against Panamanian viral strains that were isolated and previously characterized by WGS. Additionally, an RSV‐A strain (NCBI Reference Sequence: NC_038235.1) and an RSV‐B strain (MF443150.1) were included as controls.

The assay was performed in two replicates, in which various dilutions of the mAbs in DMEM supplemented with 2% inactivated FCS were incubated with 100 focus‐forming units (FFUs) of RSV isolates at room temperature for 30 min. Then, 150 μL of these mixtures was used to infect 5 × 10^4^ HEp‐2 cells in 96‐well plates. After 48 h at 37 °C with 5% CO_2_, the supernatant was removed, and the cells were fixed with cold methanol and 2% H_2_O_2_. For viral antigen detection, a cocktail of murine mAbs against G protein (021/1G and 021/21G) [37] and F protein (47F, 101F, 56F, and 2F) [38, 39] was used. The labeled FFUs were stained using an immunohistochemistry assay with HRP‐conjugated goat anti‐mouse IgG (1:500 in 1% BSA) as the secondary antibody. For visualization, 750 μL of 3‐amino‐9‐ethylcarbazole (AEC) was used as the substrate (20 mg of AEC, Sigma‐Aldrich; dissolved in 6 mL of 100% DMSO), prepared in 12 mL of 0.05 M citrate–phosphate buffer with 25 μL of 30% H₂O₂. The reaction was incubated for at least 2 h at room temperature, protected from direct light. Finally, the FFUs were counted under an inverted microscope.

IC_50_ values were calculated by fitting experimental data to a four‐parameter sigmoidal equation (4PL) using Microsoft Excel [40] (supplemental material).

## 3 Results

### 3.1 Epidemiology of RSV in Panama From 2018 to 2024

From January 2018 to July 2024, Panama Epidemiological Surveillance System detected 5702 cases of RSV infection in patients of all ages, representing 39.5% of the viruses identified during this period, excluding SARS‐CoV‐2 cases (Figure 1). The male‐to‐female ratio among these cases was 58.1%, and the ratio of hospitalized to outpatient cases was 67.0%, including one fatal case. Age distribution of cases was as follows: <1 year (55.8%), 1–4 years (35.6%), 5–50 years (6.6%), and > 50 years (1.3%) (Table 1). A total of 56.4% of the samples came from the metropolitan area and its periphery (MA&P), which in this study includes the districts of Panama, San Miguelito, Arraijan, and La Chorrera (Table 1). These four districts are among the most populated in the country and share a close social dynamic characterized by constant exchange due to commerce, work, and education [41].

**TABLE 1:** RSV‐positive samples by subgroup (A and B), and patient characteristics during the epidemic outbreaks from 2018 to 2024 in Panama.

	RSV—epidemic outbreaks														
	2018		2019–2020		2021		2022		2023		2024		Total RSV‐A	Total RSV‐B	Total (%)
	A	B	A	B	A	B	A	B	A	B	A	B			
Total	10	15	56	20	0	15	60	5	31	33	51	7	208	95	303 (100)
Gender															
Male	6	6	31	9	0	10	34	2	19	24	30	5	120	56	176 (58.1)
Female	4	9	25	11	0	5	26	3	12	8	21	2	88	38	126 (41.6)
N/A	0	0	0	0	0	0	0	0	0	1	0	0	0	1	1 (0.3)
Clinical status*															
Hospitalized	10	15	53	17	0	4	7	2	21	18	48	7	139	63	202 (66.7)
Outpatient	0	0	3	2	0	11	53	3	9	13	3	0	68	29	97 (32.0)
Deceased	0	0	0	1**	0	0	0	0	0	0	0	0	0	1**	1 (0.3)
N/A	0	0	0	0	0	0	0	0	1	2	0	0	1	2	3 (1.0)
Patient age*, years															
<1	9	13	38	16	0	4	28	2	14	15	27	3	116	53	169 (55.8)
1–4	1	2	17	4	0	8	24	3	9	14	22	4	73	35	108 (35.6)
5–50	0	0	1	0	0	2	6	0	6	3	2	0	15	5	20 (6.6)
>50	0	0	0	0	0	1	0	0	2	1	0	0	2	2	4 (1.3)
N/A	0	0	0	0	0	0	2	0	0	0	0	0	2	0	2 (0.7)
Geographic area															
MA&P	5	5	17	6	0	9	50	4	21	10	38	6	131	40	171 (56.4)
Rest of the country	5	10	39	14	0	6	10	1	10	23	13	1	77	55	132 (43.6)

*Note:* Number of samples analyzed by RT‐PCR for each epidemic outbreak studied: 2018 (*N* = 25), 2019–20 (*N* = 76), 2021 (*N* = 15), 2022 (*N* = 65), 2023 (*N* = 64), 2024 (*N* = 58).

*Abbreviation:* RSV, respiratory syncytial virus; A, RSV subgroup A; B, RSV subgroup B; N/A, information not available; MA&P, metropolitan area and its periphery.

* In 2021 and 2022, the majority of recorded patients were outpatient cases, including some individuals older than 5 years old likely due to the focus on COVID‐19 diagnostics, with samples primarily from ILI sentinel units. However, the RSV surveillance has been historically biased toward the hospitalized pediatric population.

** Six‐month‐old patient; no further information about clinical status or comorbidities could be obtained.

Respiratory virus incidence in Panama is recorded year‐round, with influenza virus, RSV, rhinovirus, and, more recently, SARS‐CoV‐2 representing the predominant circulating pathogens (Figure 1). RSV case detection typically increases during the rainy season, which spans the second half of the year (Figure 1). However, in 2020, RSV circulation was disrupted due to the implementation of strict mitigation measures to contain the spread of SARS‐CoV‐2, including the use of face masks, social distancing, and school closures. The samples collected in 2020 were detected during the first 3 months of the year, prior to the onset of the COVID‐19 pandemic, and are therefore linked to the 2019–2020 RSV outbreak (Figure 1). However, in 2021, the relaxation of COVID‐19 mitigation measures led to a resurgence of RSV cases, with detection occurring throughout the entire year (Table 1). The subset of samples that underwent genome sequencing (Ct < 28) did not differ in demographic characteristics from the original cohort (Figure 1) (Table S1).

### 3.2 Predominance and Dynamics of Subgroups and Lineages

From 2018 to 2024, the RSV‐A and the RSV‐B subgroups alternated the predominance in the epidemic outbreaks in Panama (Figure 1). However, in 2021, the number of samples analyzed was limited, and all of them corresponded to RSV‐B. We found no association between subgroups and patient gender, clinical status, or age (Fisher's exact test, *p* > 0.1). However, we found an association between subgroups and geographic zones (Fisher's exact test, *p* = 0.0011), which was driven by the cases during 2023 (Fisher's exact test, *p* = 0.0056), suggesting that there was an opposite subgroup predominance in MA&P region compared with the rest of the country in the 2023 season. In this epidemic outbreak, 67.7% of RSV‐A cases were concentrated in MA&P, whereas similarly, 69.7% of RSV‐B cases were located in the rest of the country.

**FIGURE 2:**
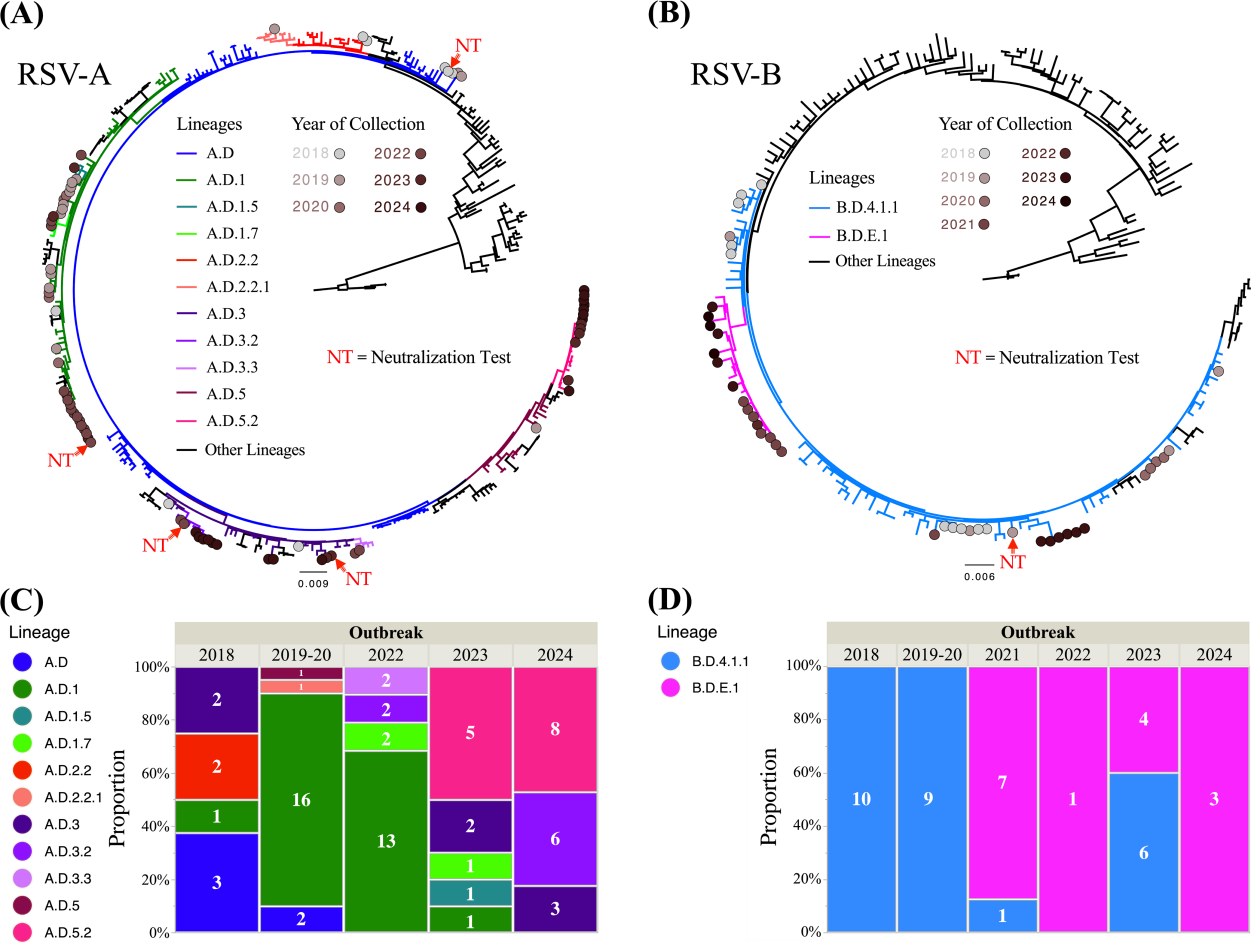
Maximum likelihood phylogenetic trees of the complete genomes of RSV‐A (A) and RSV‐B (B) grouped with reference strains. The RSV genomes from Panama are highlighted with circles ranging from light gray (2018) to dark red (2024) at the tips of the branches. The RSV subgroups are represented by the colors of the tree branches. The scale bar indicates nucleotide substitutions per site. Inside the box, stacked bar graphs for RSV‐A (C) and RSV‐B (D) represent the RSV lineages identified during the study year, with the label on each bar corresponding to the number of genomes obtained. Maximum likelihood phylogenetic trees based on the Fusion protein gene are presented in Figure S1. The graph was plotted in Jump Statistical Software 18.0.1. RSV‐A indicates respiratory syncytial virus subgroup A; RSV‐B, respiratory syncytial virus subgroup B.

Phylogenetic analysis of subgroup A genomes identified 11 distinct RSV‐A lineages, with evidence of cocirculation of three to five lineages during each annual outbreak (Figure 2).

Several RSV‐A lineages detected prior to the COVID‐19 pandemic, including a.d.2.2, a.d.2.2.1, and a.d.5, were not identified in samples collected after 2020. In turn, subgroup B phylogenetic analysis showed the circulation of two RSV‐B lineages: B.D.4.1.1 in 2018 and 2019–2020, and since 2021, the descendant lineage B.D.E.1 was detected cocirculating and prevailing to B.D.4.1.1 (Figure 2).

**FIGURE 3:**
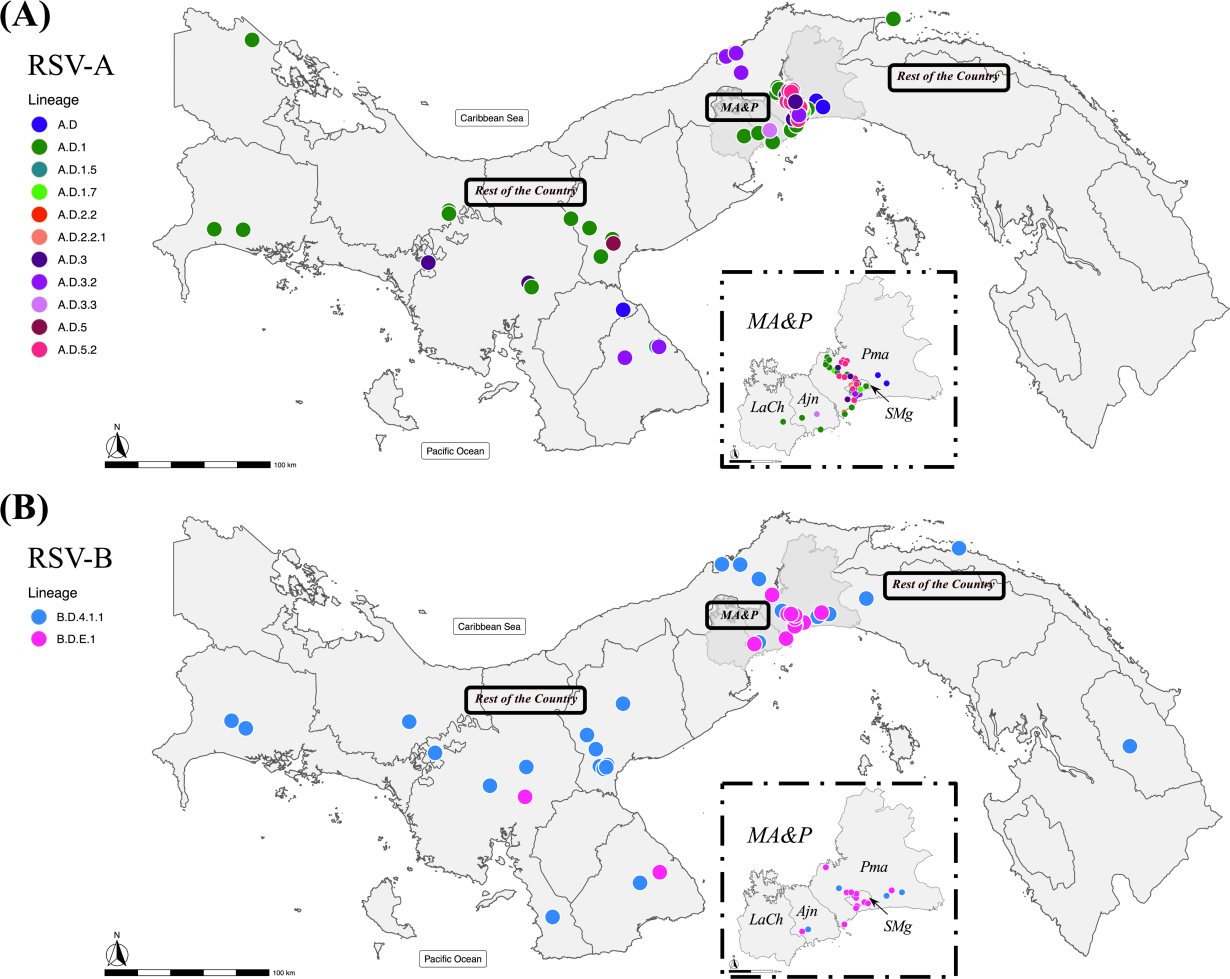
A geographic map of Panama is presented showing the location of the lineages found for RSV‐A (A) and RSV‐B (B) in the epidemic outbreaks studied. The map was generated using R software 4.1.0. RSV‐A, respiratory syncytial virus subgroup A; RSV‐B, respiratory syncytial virus subgroup B; MA&P, metropolitan area and its periphery; Pma, Panama; SMg, San Miguelito; Ajn, Arraijan; LCh, La Chorrera.

Geographical analysis of lineage distribution revealed that lineages a.d.1.7, a.d.3.2, a.d.3.3, a.d.5.2, and B.D.E.1 were initially identified in the metropolitan area and Panama (MA&P) (Figure 3) (Table S2), which is the most densely populated area. This area may serve as a focal point for the introduction and subsequent spread of the lineages. We found geographically limited transmission for some lineages within RSV‐A and RSV‐B lineages (chi‐square test, *p* = 0.033 and *p* = 0.0001, respectively) (Table S3).

The 13 RSV lineages found in the study were detected in children under 5 years of age, but only five lineages in individuals older than 5 years old (Table S4). For RSV‐A lineages, the chi‐square test did not reveal a significant association with hospitalization (*p* = 0.091; Table S3). Within the RSV‐B subgroup, the B.D.4.1.1 lineage was predominantly associated with hospitalized cases (Fisher's exact test, *p* = 0.004) and was also detected in the single deceased patient, whereas the B.D.E.1 lineage was more frequently observed in outpatient cases.

### 3.3 Sensitivity of Current RSV Strains to Monoclonal Antibodies

To assess the sensitivity of RSV strains detected in Panama to mAbs MEDI8897*, motavizumab, and AM14, we successfully isolated 18 RSV‐A from different lineages (one A.D., thirteen a.d.1, three a.d.3, and one a.d.3.2) and one RSV‐B (B.D.4.1.1). Unfortunately, it was not possible to obtain viable isolates from all detected lineages, which limited the scope of the neutralization assays, also representing a pending aspect that should be addressed in future studies. The RT‐qPCR Ct values of the NPS from which viral isolation was achieved ranged between 16.8 and 24.8, with a median of 20.8 (Table S5). We aimed to include strains representing the greatest genetic diversity; however, due to the inherent limitations of RSV isolation, only representative strains from clades A. D, a.d.1, a.d.3.2, a.d.3, and B.D.4.1.1 could be obtained for further analysis. The selected strains were hRSV/A/Panama/ICGES‐215/2018 (A.D), hRSV/A/Panama/ICGES‐45/2022 (a.d.1), hRSV/A/Panama/ICGES‐42/2022 (a.d.3.2), hRSV/A/Panama/ICGES‐147/2023 (a.d.3), and hRSV/B/Panama/ICGES‐114/2019 (B.D.4.1.1) (Figure 2). All five isolates exhibited significant sensitivity to the mAbs, but especially to MEDI8897* with an IC_50_ range of 0.30–6.99 ng/mL, 6.56–14.97 ng/mL for AM14, and 13.15–69.79 ng/mL for motavizumab (Table 2) (Figure S2).

**TABLE 2:** Determination of IC_50_ of mAbs (ng/mL) targeting the F protein of RSV in Panamanian RSV strains isolated from 2018 to 2023.

Strain name	Monoclonal antibodies (mAbs)		
	MEDI8897*	AM14	MOTAVIZUMAB
hRSV/A/Panama/ICGES‐215/2018 (A.D.)	2.55	6.56	13.15
hRSV/A/Panama/ICGES‐45/2022 (a.d.1)	6.99	11.19	35.30
hRSV/A/Panama/ICGES‐42/2022 (A.D.3.2)	6.41	10.27	69.79
hRSV/A/Panama/ICGES‐147/2023 (A.D.3)	5.85	14.97	49.85
hRSV/B/Panama/ICGES‐114/2019 (B.D.4.1.1)	0.30	12.10	41.66
hRSV/A/A2 strain (control)	1.22	7.00	25.84
hRSV/B/GM3.5/Spain (control)	3.15	4.52	35.87

*Note:* RSV lineage for each strain is detailed between parentheses. The statistical analysis of the Panamanian isolates showed that MEDI8897* had an IC_50_ (ng/mL) average of 4.42 ng/mL (95% confidence interval [CI], 0.85–7.99). In turn, AM14 showed an IC_50_ of 11.02 ng/mL (95% CI, 7.23–14.81), and motavizumab an IC_50_ of 41.95 ng/mL (95% CI, 16.27–67.63). The irrelevant control MF14 (anti‐F metapneumovirus) showed an IC_50_ > 1666 ng/mL. Control strains included were RSV‐A A2 (NC_038235.1, genotype A2, lacking the 72‐nt insertion in the G gene) and RSV‐B GM3.5/Spain (MF443150.1, genotype BA, carrying the 60‐nt insertion in the G gene).

*Abbreviation:* IC_50_, half‐maximal inhibitory concentration.

Interestingly, RSV‐B hRSV/B/Panama/ICGES‐114/2019 (lineage B.D.4.1.1) demonstrated the highest sensitivity to MEDI8897*, with an IC_50_ of 0.30 ng/mL. RSV‐A strains showed slight variability in sensitivity to MEDI8897*, with IC_50_ values ranging from 2.55 ng/mL for hRSV/A/Panama/ICGES‐215/2018 (A.D) to 6.99 ng/mL for hRSV/A/Panama/ICGES‐45/2022 (a.d.1) (Table 2).

A detailed analysis of the F protein sequence in isolated RSV‐A strains revealed amino acid substitutions at the binding sites compared with the NCBI GenBank reference strain (accession number NC_038235). No substitutions were observed in the nirsevimab binding site. In the motavizumab binding site, the strains hRSV/A/Panama/ICGES‐215/2018, hRSV/A/Panama/ICGES‐45/2022, and hRSV/A/Panama/ICGES‐147/2023 showed the N276S substitution. By contrast, the strain hRSV/A/Panama/ICGES‐42/2022 regained the asparagine at this position, highlighting its importance as a lineage‐defining amino acid [8]. In the clersovimab binding site, which is similar to that of AM14, all RSV‐A strains shared the M447V substitutions. Furthermore, multiple substitutions were identified in other regions of the F protein, including L4P, A8T, T16A, F20L, G25S, P102A, T103A, N105S, A122T, K124N, V139G, V152I, L178V, I379V, and S540A. Moreover, T12I was detected in the strains hRSV/A/Panama/ICGES‐42/2022 and hRSV/A/Panama/ICGES‐147/2023, underscoring its relevance in this lineage (lineage‐defining amino acid) [8]. A comparison of these findings with current GISAID reference strains is provided in Table S5.

On the other hand, the RSV‐B strain hRSV/B/Panama/ICGES‐114/2019 exhibited a distinct pattern of amino acid substitutions when analyzed against the NCBI GenBank reference strain (accession number NC_001781). In the nirsevimab binding site, the substitutions I206M and Q209R were identified, whereas no changes were observed in the motavizumab binding site or the clersovimab binding site. In other regions of the F protein, additional substitutions were detected, including L8S, F45L, A103V, L172Q, S173L, K191R, N234T, and T529A, which could be associated with specific adaptations of this strain. The amino acid sequence of the analyzed Panamanian RSV‐B strain is 100% homologous to the F protein of the hRSV/B/Australia/VIC‐RCH056/2019 strain (EPI_ISL_1653999), with the latter being the EpiRSV reference (GISAID) (Table S5).

## 4 Discussion

This study offers a detailed overview of RSV genotypic and phenotypic characteristics in Panama over the last 7 years. Our findings reveal alternating predominance between RSV‐A and RSV‐B subgroups, the emergence and cocirculation of diverse lineages, and high sensitivity of current RSV strains to monoclonal antibodies, including MEDI8897*, a nirsevimab parental. Our study underscores the RSV epidemiology and potential responses to emerging prevention strategies, particularly as Panama is a critical global trade hub and tourism destination, which may affect viral introductions and circulation.

The National Epidemiological Surveillance System has recorded, between 2018 and 2024, a significant incidence of cases attributed to RSV, with clearly identified annual epidemic outbreaks. These outbreaks have historically been associated with the rainy season in tropical climates [42], which is consistent with the climate of our country and with the findings of this study. In March 2020, following the confirmation of the first COVID‐19 case in Panama, the government implemented strict containment measures, such as school closures, social distancing, and curfews [43]. As also happened in other countries, these interventions had the additional effect of disrupting the transmission of other respiratory viruses, except for rhinovirus [44, 45]. However, RSV resurged in Panama in 2021, with year‐long detections, as was also reported in other regions [46, 47]. This shows the sensitivity of RSV epidemiology to changes in social behavior and highlights the importance of maintaining an active viral surveillance systems, even in the context of pandemic‐related disruptions. The metropolitan area of Panama City, a densely populated region and a focal point for international travel through its airport and the Panama Canal, showed the most diverse RSV circulation, suggesting a connection between population density, travel, and viral introductions. Comparable patterns have been documented in urban centers worldwide, where newly emerging RSV lineages are typically introduced in densely populated urban areas prior to dissemination into peripheral or less populated regions [48]. This observation also aligns with previous studies suggesting that RSV transmission and geographic spread are predominantly driven by air travel [49].

We observed an alternating pattern of annual subgroup replacement between RSV‐A and RSV‐B. This phenomenon has been documented in other regions of the world [50, 51]. Such subgroup replacement patterns may be attributed to antigenic drift inducing viral fitness, or fluctuations in population immunity following previous outbreak [25]. Our study found that RSV‐B predominated in 2021, likely due to the introduction the B.D.E.1 lineage. The predominance would be also favored by reduced viral exposure during the pandemic [52, 53]. Conversely, in 2022, RSV‐A became the predominant subgroup. This shift may be linked to herd immunity from prior RSV‐B outbreak [25]. Notably, different patterns have been reported in other regions of the world: in Europe, RSV‐A predominated in 2021, whereas RSV‐B predominated during the 2022/2023 epidemic [54, 55, 56]. However, in New Zealand, immediately after the pandemic (2021), both subgroups re‐emerged simultaneously without a clear predominance [10]. These observations, in concordance with previous reports [24], highlight that the distribution of RSV subgroups may vary according to the region.

Overall, our observations regarding the lineages detected in the last six epidemic outbreaks in Panama are consistent with reports globally [11, 57, 58, 59]. These studies have documented that the reintroduction of RSV following preventive measures against COVID‐19 showed a gradual recovery of transmission patterns, with a postpandemic predominance or significant presence of the a.d.1 and ad.5.2 lineages in RSV‐A, and B.D.4.1.1 and B.D.E.1 in RSV‐B. Moreover, our study showed that RSV‐A had greater genetic diversity than RSV‐B, as evidenced by the higher number of cocirculating lineages. This observation aligns with reports of RSV‐A exhibiting a substantially greater variety of lineages in circulation compared with RSV‐B across multiple countries over several years [49].

In addition, an association was observed between the RSV‐B lineage B.D.4.1.1 and increased hospitalization rates, including its identification in a fatal case of RSV infection. Although more research is needed to definitively link RSV lineages with disease severity, studies have found that specific postpandemic RSV strains have been associated with greater clinical severity [55, 60]. Therefore, understanding these associations between RSV subgroups driven by different lineages is essential for anticipating public health burdens in future RSV seasons and developing targeted interventions.

The sensitivity of RSV strains to three monoclonal antibodies—MEDI8897* (parental nirsevimab), motavizumab, and AM14—was also evaluated. Promisingly, all tested RSV strains, regardless of lineage, were highly sensitive to these antibodies. Additionally, the IC_50_ values obtained in our study are very similar and comparable with those reported in previous studies, supporting the high potency of these antibodies in neutralizing the virus [20]. Although strains with up to 20 substitutions in the F protein compared with the reference strain were identified (Table S5), the antigenic sites targeted by the studied mAbs remained highly conserved. This is consistent with a previous report indicating that, between 2015 and 2021, the nirsevimab binding site remained highly conserved in RSV‐A (100%) and in 88% of the positions in RSV‐B. Similarly, the key antigenic sites (Ø and II) remained stable throughout the entire study period (1956–2021), highlighting the relevance of these sites in the efficacy of monoclonal antibodies [61]. Notably, the hRSV/B/Panama/ICGES‐114/2019 strain from the lineage B.D.4.1.1 showed the highest sensitivity to nirsevimab, with an IC_50_ of 0.30 ng/mL. This high susceptibility is attributable to the I206M and Q209R substitutions located in antigenic site ⌀ of the F protein. Recent studies suggest that these substitutions enhance electrostatic interactions between the antigenic site and nirsevimab, significantly improving its viral neutralization capacity [62]. The fact that lineages such as B.D.4.1.1, which were associated with increased hospitalizations and a fatal case in our study, are highly susceptible to nirsevimab suggests that susceptibility to monoclonal antibodies may be independent of any potential correlation with disease severity. However, it is important to highlight that the B.D.4.1.1 lineage detected in this study in 2023 once again has a Glutamine at position 209. Additionally, the B.D.E.1 strains present an S211N substitution (a lineage‐defining amino acid) [8]. The absence of B.D.E.1 isolates could be due to a low amount of viable virus in the clinical samples and/or specific characteristics of this lineage that may hinder its isolation. Despite these recent substitutions, previous studies confirm that RSV‐B strains remain sensitive to nirsevimab [62]. These findings suggest that the implementation of nirsevimab, or another preventive treatment with proven safety and efficacy, could be highly effective in Panama, particularly in preventing severe cases and deaths associated with RSV.

The effectiveness of these antibodies, combined with genomic surveillance, will be critical in predicting the future success of RSV vaccines and therapeutic interventions. As monoclonal antibodies and vaccines are rolled out in Panama and other regions, active viral genomic surveillance will be essential to predict their effectiveness to fight RSV, and at the same time evaluate whether and how their implementation in Panama and globally might pressure RSV evolution.

## 5 Conclusions

This study provides a comprehensive analysis of RSV evolution in Panama over the past 7 years. Our results reveal considerable genetic diversity in RSV‐A compared with RSV‐B, with the cocirculation of multiple lineages in each annual outbreak. In addition, the impact of the COVID‐19 pandemic on RSV circulation in 2020 is evident, along with atypical seasonality in 2021 and the emergence and replacement of lineages in subsequent years. All tested RSV strains were neutralized by monoclonal antibodies, especially by the parental antibody of nirsevimab, which highlights the potential of these treatments to reduce RSV disease severity. However, the interpretation of these findings should consider certain study limitations, particularly the restricted availability of clinical samples and the challenges in obtaining isolates representative of all circulating lineages. Continuous surveillance and molecular characterization of these strains are essential, especially when preventive interventions are implemented, and, in a hypothetical worst‐case scenario, variants with immune escape mechanisms could emerge.

## Author Contributions

**Danilo Franco:** investigation, methodology, data curation, visualization, writing – original draft, conceptualization. **Stephanie Goya:** validation, methodology, formal analysis, visualization, writing – review and editing. **Alexander Martínez:** validation, methodology, formal analysis, visualization, writing – review and editing. **Vicente Mas:** investigation, writing – review and editing, resources, methodology, supervision. **Brechla Moreno:** investigation, resources, investigation, resources, investigation, resources. **Elimelec Valdespino:** investigation, resources. **Melissa Gaitán:** investigation, resources. **Lisseth Sáenz:** investigation, resources. **Claudia González:** investigation, resources. **Ambar Moreno:** investigation, resources, investigation, resources, investigation, resources. **Zeuz Capitan‐Barrios:** validation, project administration, visualization, writing – review and editing. **Jean Paul Carrera:** validation, formal analysis, writing – review and editing. **Sandra López‐Vergès:** validation, writing – review and editing, formal analysis. **Juan Miguel Pascale:** investigation, writing – review and editing, resources. **Yadira Moltó:** investigation, resources. **Lourdes Moreno:** investigation, resources, investigation, resources, investigation, resources. **Belmaris Rizo:** investigation, resources. **Enrique Urriola:** investigation, resources. **Teresa Delgado:** investigation, resources. **María Iglesias‐Caballero:** investigation, resources. **Inmaculada Casas:** supervision, resources. **Suman R. Das:** investigation, resources, resources, investigation. **Juan Arbiza:** supervision, writing – review and editing. **Adriana Delfraro:** supervision, resources, writing – review and editing, validation, visualization. **Leyda Ábrego:** conceptualization, funding acquisition, supervision, methodology, formal analysis, visualization, writing – review and editing.

## Conflicts of Interest

The authors declare that the research was conducted in the absence of any commercial or financial relationships that could be construed as a potential conflicts of interest.

## Peer Review

The peer review history for this article is available at https://www.webofscience.com/api/gateway/wos/peer‐review/10.1111/irv.70173.

## Supporting information


**Table S1:** RSV genomes obtained, distributed by epidemic outbreak (2018 to 2024), antigenic subgroup, and demographic characteristics of patients in Panama.
**Table S2:** Clinical and demographic characteristics of RSV‐positive cases (2018–2024) sequenced by WGS and grouped by epidemic outbreak and lineage found.
**Table S3:** Statistical Association Between RSV Lineages and Demographic Characteristics Using Chi‐Square Test of Independence.
**Table S4:** RSV lineages involved in the 2018–2024 epidemic outbreaks based on the clinical and demographic characteristics of the patients.
**Table S5:** Information about the RSV strains isolated and those selected for the neutralization assay with mAbs.
**Figure S1:** Maximum likelihood phylogenetic trees of Fusion gen of RSV‐A and RSV‐B grouped with reference strains. RSV genomes from Panama are highlighted with circles ranging from light red (2018) to dark red (2024) at branch tips. RSV subgroups are represented by the colors of the tree branches. The isolated strains subjected to neutralization assays (NT) against the F protein are indicated in the tree. The scale bar indicates nucleotide substitutions per site. RSV‐A, RSV subgroup A; RSV‐B, RSV subgroup B; NT, neutralization test.
**Figure S2:** Determination of IC50 values for RSV isolates. Each graph corresponds to a specific strain, indicated at the top of each plot. Neutralization assays were performed using monoclonal antibodies (mAbs), with their respective concentrations (ng/mL) shown on the x‐axis. The y‐axis represents the number of FFU counted in each well. mAbs are color coded as follows: MEDI8897* (turquoise), AM14 (magenta), motavizumab (blue), and MF 14 (green). IC50 values were calculated by fitting the experimental data to a four‐parameter logistic (4PL) equation using Microsoft Excel (Sebaugh, 2011). hRSV, human respiratory syncytial virus; FFU, focus‐forming units; mAbs, monoclonal antibodies; ng, nanograms; mL, milliliters.

## Data Availability

RSV whole genomes from this study are available in EpiRSV (GISAID) (accession numbers EPI_ISL_19423081‐EPI_ISL_19423195) and NCBI GenBank (accession numbers PQ618008‐PQ618122).
