# Supplementary material for: Genomic Insights Into Respiratory Syncytial Virus Circulation Patterns and Neutralization by Anti‐F Monoclonal Antibodies in Panama (2018–2024)
Source: Influenza Other Respir Viruses. 2025 Oct 22;19(10):e70173. doi: 10.1111/irv.70173 (PMC12545694; doi:10.1111/irv.70173)
Supplement: Supplementary file 1 — Table S1: RSV genomes obtained, distributed by epidemic outbreak (2018 to 2024), antigenic subgroup, and demographic characteristics of patients in Panama. Table S2: Clinical and demographic characteristics of RSV‐positive cases (2018–2024) sequenced by WGS and grouped by epidemic outbreak and lineage found. Table S3: Statistical Association Between RSV Lineages and Demographic Characteristics Using Chi‐Square Test of Independence. Table S4: RSV lineages involved in the 2018–2024 epidemic outbreaks based on the clinical and demographic characteristics of the patients. Table S5: Information about the RSV strains isolated and those selected for the neutralization assay with mAbs. Figure S1: Maximum likelihood phylogenetic trees of Fusion gen of RSV‐A and RSV‐B grouped with reference strains. RSV genomes from Panama are highlighted with circles ranging from light red (2018) to dark red (2024) at branch tips. RSV subgroups are represented by the colors of the tree branches. The isolated strains subjected to neutralization assays (NT) against the F protein are indicated in the tree. The scale bar indicates nucleotide substitutions per site. RSV‐A, RSV subgroup A; RSV‐B, RSV subgroup B; NT, neutralization test. Figure S2: Determination of IC50 values for RSV isolates. Each graph corresponds to a specific strain, indicated at the top of each plot. Neutralization assays were performed using monoclonal antibodies (mAbs), with their respective concentrations (ng/mL) shown on the x‐axis. The y‐axis represents the number of FFU counted in each well. mAbs are color coded as follows: MEDI8897* (turquoise), AM14 (magenta), motavizumab (blue), and MF 14 (green). IC50 values were calculated by fitting the experimental data to a four‐parameter logistic (4PL) equation using Microsoft Excel (Sebaugh, 2011). hRSV, human respiratory syncytial virus; FFU, focus‐forming units; mAbs, monoclonal antibodies; ng, nanograms; mL, milliliters. [file IRV-19-e70173-s001.pdf]

Supplemental Material

List of Two hundred seventy-seven (277) complete reference genomes of RSV-A subgroup, downloaded from the RSV Genotyping Consensus Consortium (https://github.com/rsv-lineages). Data obtained on August 26, 2024.

1. A.1/MG642060/USA/1980
2. A.1/MG642067/USA/1987
3. A.1/MG642075/USA/1986
4. A.1/OK649589/USA/1994
5. A.1/OK649593/USA/1995
6. A.2.1.1/KF973325/USA/2002
7. A.2.1.1/KF973335/USA/2002
8. A.2.1.1/KJ627716/USA/2003
9. A.2.1.1/KJ627719/USA/2004
10. A.2.1.1/KJ627721/USA/2004
11. A.2.1.1/OK649606/Brazil/2008
12. A.2.1.1/OK649637/Brazil/2009
13. A.2.1.1/OK649646/Brazil/2010
14. A.2.1/KJ627657/USA/2003
15. A.2.1/KJ627671/USA/2003
16. A.2.1/KJ627673/USA/2001
17. A.2.1/KJ627705/USA/2001
18. A.2.1/KJ627710/USA/2001
19. A.2.1/KJ627733/USA/2003
20. A.2/MG642028/USA/1980
21. A.2/MG642052/USA/1994
22. A.2/MG642055/USA/1990
23. A.2/MG642058/USA/1980
24. A.2/MG642079/USA/1982
25. A.3.1.1/KJ627738/USA/2013
26. A.3.1.1/KY654506/Philippines/2012
27. A.3.1.1/KY654508/Philippines/2012
28. A.3.1.1/MF001043/USA/2015
29. A.3.1.1/OK649639/Brazil/2009
30. A.3.1.1/OK649647/Brazil/2010
31. A.3.1.1/ON237251/Argentina/2015
32. A.3.1.1/ON237255/Argentina/2015
33. A.3.1/MF001041/USA/2015
34. A.3.1/MH181883/Kenya/2012
35. A.3.1/MK109777/Jordan/2011
36. A.3.1/OK649604/Brazil/2008
37. A.3.1/OK649610/Brazil/2008
38. A.3.1/OK649623/Brazil/2008
39. A.3.1/OK649628/Brazil/2008
40. A.3.1/OK649633/Brazil/2008
41. A.3.1/OK649645/Brazil/2010
42. A.3/KJ627647/USA/2001
43. A.3/MG642026/USA/1982
44. A.3/MG642033/USA/1994
45. A.3/MG642034/USA/1985
46. A.3/MG642035/USA/1984
47. A.3/MG642070/USA/1986
48. A.3/MG642080/USA/1994
49. A.3/OK649614/Brazil/2008
50. A.3/OK649627/Brazil/2008
51. A.3/OK649642/Brazil/2010
52. A.3/OK649678/USA/2005
53. A.3/OK649679/USA/2005
54. A.D.1.1/EPI-ISL-11817023/Australia/2021
55. A.D.1.1/EPI-ISL-11817069/Australia/2021
56. A.D.1.1/EPI-ISL-11817092/Australia/2021
57. A.D.1.2/OM857140/Australia/2021
58. A.D.1.2/OM857152/Australia/2020
59. A.D.1.2/OM857155/Australia/2020
60. A.D.1.2/OM857177/Australia/2021
61. A.D.1.2/OM857180/Australia/2020
62. A.D.1.2/OM857200/Australia/2020
63. A.D.1.3/OM857216/Australia/2020
64. A.D.1.3/OM857267/Australia/2021
65. A.D.1.3/OM857271/Australia/2020
66. A.D.1.3/OM857282/Australia/2020
67. A.D.1.3/OM857283/Australia/2020
68. A.D.1.3/OM857312/Australia/2020
69. A.D.1.3/OM857320/Australia/2020
70. A.D.1.3/OM857360/Australia/2021
71. A.D.1.4/OQ024155/USA/2022
72. A.D.1.4/OR143137/USA/2022
73. A.D.1.4/OY757595/Australia/2022
74. A.D.1.4/PP352344/USA/2022
75. A.D.1.4/PP681283/USA/2022
76. A.D.1.4/PP770472/USA/2024
77. A.D.1.5/OQ171906.1/USA/2022
78. A.D.1.5/OR143171.1/USA/2023
79. A.D.1.6/PP135017.1/USA/2023
80. A.D.1.6/PP352345.1/USA/2022
81. A.D.1.6/PP795136.1/USA/2023
82. A.D.1.7/OQ171911.1/USA/2022
83. A.D.1.7/PP203260.1/USA/2022
84. A.D.1.8/OR287988.1/USA/2021
85. A.D.1.8/PP352324.1/USA/2021
86. A.D.1.8/PP973760.1/USA/2024
87. A.D.1/MZ221198/China/2018
88. A.D.1/MZ515573/United-Kingdom/2020

89. A.D.1/MZ515583/Spain/2019
90. A.D.1/MZ515649/Netherlands/2019
91. A.D.1/MZ515773/Spain/2020
92. A.D.1/MZ515777/Spain/2019
93. A.D.1/MZ515803/Netherlands/2019
94. A.D.1/MZ515907/United-Kingdom/2019
95. A.D.1/MZ515929/United-Kingdom/2018
96. A.D.1/MZ515931/Netherlands/2019
97. A.D.1/MZ515945/Netherlands/2018
98. A.D.1/MZ515961/Spain/2020
99. A.D.1/MZ515969/United-Kingdom/2019
100. A.D.1/MZ516000/United-Kingdom/2019
101. A.D.1/MZ516029/United-Kingdom/2019
102. A.D.1/MZ516033/Spain/2020
103. A.D.1/MZ516040/United-Kingdom/2019
104. A.D.1/MZ516058/Netherlands/2017
105. A.D.1/MZ516073/United-Kingdom/2019
106. A.D.1/MZ516137/United-Kingdom/2019
107. A.D.1/ON237313/Argentina/2017
108. A.D.1/OQ171909/USA/2022
109. A.D.1/OQ261751/Austria/2022
110. A.D.2.1/MZ515701/Netherlands/2019
111. A.D.2.1/MZ515859/Netherlands/2019
112. A.D.2.1/MZ515877/Netherlands/2019
113. A.D.2.1/MZ516038/Netherlands/2019
114. A.D.2.2.1/LR699737/United-Kingdom/2017
115. A.D.2.2.1/MZ515575/United-Kingdom/2018
116. A.D.2.2.1/MZ515609/Spain/2019
117. A.D.2.2.1/MZ515850/United-Kingdom/2018
118. A.D.2.2.1/MZ515851/Spain/2018
119. A.D.2.2.1/MZ515956/Spain/2019
120. A.D.2.2.1/MZ516026/Netherlands/2019
121. A.D.2.2.1/ON237289/Argentina/2016
122. A.D.2.2/MH383066/Lebanon/2014
123. A.D.2.2/MK749886/Nicaragua/2016
124. A.D.2.2/MK749893/Nicaragua/2016
125. A.D.2.2/MK749911/Nicaragua/2016
126. A.D.2.2/MN630100/USA/2016
127. A.D.2.2/MZ515703/Netherlands/2018
128. A.D.2.2/MZ515835/Netherlands/2018
129. A.D.2.2/MZ515939/Netherlands/2017
130. A.D.2.2/MZ516072/United-Kingdom/2019
131. A.D.2.2/ON237253/Argentina/2015
132. A.D.2.2/ON237273/Argentina/2015
133. A.D.2.2/ON237301/Argentina/2017
134. A.D.2.2/ON237336/Argentina/2017
135. A.D.2.2/ON237340/Argentina/2017
136. A.D.2/MK749912/Nicaragua/2015
137. A.D.2/MK749913/Nicaragua/2016
138. A.D.2/MZ515901/Netherlands/2019
139. A.D.2/ON237272/Argentina/2015
140. A.D.3.1/EPI-ISL-15896149/Australia/2022
141. A.D.3.1/EPI-ISL-15896158/Australia/2022
142. A.D.3.1/EPI-ISL-15896167/Australia/2022
143. A.D.3.1/EPI-ISL-15896183/Australia/2022
144. A.D.3.1/EPI-ISL-15896222/Australia/2022
145. A.D.3.1/EPI-ISL-16714780/Australia/2022
146. A.D.3.2/OR143195.1/USA/2022
147. A.D.3.2/PP135024.1/USA/2023
148. A.D.3.2/PP508181.1/Peru/2022-05-30
149. A.D.3.3/PP530269.1/USA/2024
150. A.D.3.3/PP748752.1/USA/2021
151. A.D.3.3/PP770461.1/USA/2024
152. A.D.3.4/OQ024115.1/USA/2022
153. A.D.3.4/OQ261753.1/Austria/2022
154. A.D.3.4/OR143204.1/USA/2022
155. A.D.3.5/OR795475.1/Germany/2022
156. A.D.3.5/PP709453.1/USA/2024
157. A.D.3.6/PP237786.1/USA/2023
158. A.D.3.6/PP401817.1/USA/2023
159. A.D.3.6/PP504647.1/USA/2023
160. A.D.3/MN306029/USA/2019
161. A.D.3/MT422270/Russia/2019
162. A.D.3/MZ151853/Russia/2020
163. A.D.3/MZ515619/Spain/2019
164. A.D.3/MZ515679/United-Kingdom/2020
165. A.D.3/MZ515693/Spain/2020
166. A.D.3/MZ515731/Netherlands/2018
167. A.D.3/MZ515923/United-Kingdom/2019
168. A.D.3/MZ516023/United-Kingdom/2020
169. A.D.3/MZ516039/Netherlands/2019
170. A.D.3/MZ516110/Netherlands/2018
171. A.D.3/MZ516112/United-Kingdom/2019
172. A.D.3/OK500256/France/2021
173. A.D.3/ON152648/Russia/2021
174. A.D.3/OP890340/USA/2022
175. A.D.3/OQ261748/Austria/2021
176. A.D.4.1/EPI-ISL-11055718/South-Africa/2022
177. A.D.4.1/EPI-ISL-11055743/South-Africa/2021
178. A.D.4.1/EPI-ISL-12529638/South-Africa/2021
179. A.D.4.1/EPI-ISL-12529640/South-Africa/2021
180. A.D.4.1/EPI-ISL-12529643/South-Africa/2021
181. A.D.4.1/MZ515682/United-Kingdom/2019
182. A.D.4/MH181991/Kenya/2013
183. A.D.4/MH182003/Kenya/2014
184. A.D.4/MH182010/Kenya/2014
185. A.D.4/MH182012/Kenya/2014
186. A.D.4/MH182018/Kenya/2014

187. A.D.4/MH182019/Kenya/2014
188. A.D.4/MH182031/Kenya/2015
189. A.D.4/MH182036/Kenya/2015
190. A.D.4/MH182049/Kenya/2015
191. A.D.5.1/EPI-ISL-11055722/South-Africa/2022
192. A.D.5.1/EPI-ISL-11055747/South-Africa/2021
193. A.D.5.1/EPI-ISL-11055760/South-Africa/2021
194. A.D.5.1/MZ515749/United-Kingdom/2019
195. A.D.5.2/EPI-ISL-15055327/Argentina/2021
196. A.D.5.2/OK500258/France/2021
197. A.D.5.2/OP890318/USA/2022
198. A.D.5.2/OP890321/USA/2022
199. A.D.5.2/OP890332/USA/2022
200. A.D.5.2/OP890338/USA/2022
201. A.D.5.2/OP890339/USA/2022
202. A.D.5.2/OQ024127/USA/2022
203. A.D.5.2/OQ171903/USA/2022
204. A.D.5.3/EPI-ISL-16959591/New-Zealand/2021
205. A.D.5.3/EPI-ISL-16959666/New-Zealand/2021
206. A.D.5.3/EPI-ISL-16959728/New-Zealand/2021
207. A.D.5.3/EPI-ISL-16959943/New-Zealand/2021
208. A.D.5.3/EPI-ISL-16960085/New-Zealand/2021
209. A.D.5.3/EPI-ISL-16960101/New-Zealand/2021
210. A.D.5.4/PP135013.1/USA/2023
211. A.D.5.4/PP237781.1/USA/2023
212. A.D.5/EPI-ISL-15055318/Argentina/2019
213. A.D.5/EPI-ISL-15120672/Morocco/2014
214. A.D.5/MZ515616/Netherlands/2019
215. A.D.5/MZ515782/United-Kingdom/2018
216. A.D.5/MZ515884/Netherlands/2019
217. A.D.5/ON237271/Argentina/2015
218. A.D.5/ON237320/Argentina/2017
219. A.D.5/OP320383/Philippines/2019
220. A.D.5/OP320385/Philippines/2019
221. A.D.5/OP320394/Philippines/2019
222. A.D.5/OP320399/Philippines/2020
223. A.D/KJ627735/USA/2013
224. A.D/KY654513/Philippines/2013
225. A.D/KY654515/Philippines/2012
226. A.D/KY982516/USA/2013
227. A.D/MF614946/China/2012
228. A.D/MF614947/China/2013
229. A.D/MH181911/Kenya/2012
230. A.D/MH181917/Kenya/2012
231. A.D/MH181927/Kenya/2012
232. A.D/MH181931/Kenya/2012
233. A.D/MH181938/Kenya/2012
234. A.D/MH181940/Kenya/2012
235. A.D/MH181941/Kenya/2012
236. A.D/MH181945/Kenya/2012
237. A.D/MH181947/Kenya/2012
238. A.D/MH181948/Kenya/2012
239. A.D/MH181960/Kenya/2012
240. A.D/MH181969/Kenya/2012
241. A.D/MH181974/Kenya/2012
242. A.D/MH181982/Kenya/2013
243. A.D/MH181985/Kenya/2013
244. A.D/MH279547/China/2017
245. A.D/MH447953/Thailand/2013
246. A.D/MH447956/Thailand/2014
247. A.D/MH447957/Thailand/2016
248. A.D/MN630090/USA/2016
249. A.D/MN630093/USA/2016
250. A.D/MN630106/USA/2016
251. A.D/MW020596/Australia/2018
252. A.D/MW020598/Australia/2017
253. A.D/MZ221197/China/2018
254. A.D/MZ221199/China/2018
255. A.D/MZ515600/United-Kingdom/2018
256. A.D/MZ515802/United-Kingdom/2019
257. A.D/MZ515913/Netherlands/2019
258. A.D/MZ515973/Netherlands/2017
259. A.D/MZ516057/Netherlands/2019
260. A.D/MZ516076/United-Kingdom/2018
261. A.D/OK649682/USA/2012
262. A.D/ON237225/Argentina/2014
263. A.D/ON237229/Argentina/2014
264. A.D/ON237230/Argentina/2014
265. A.D/ON237243/Argentina/2014
266. A.D/ON237250/Argentina/2015
267. A.D/ON237252/Argentina/2015
268. A.D/ON237254/Argentina/2015
269. A.D/ON237258/Argentina/2015
270. A.D/ON237259/Argentina/2015
271. A.D/ON237276/Argentina/2016
272. A.D/ON237277/Argentina/2016
273. A.D/ON237280/Argentina/2016
274. A.D/ON237286/Argentina/2016
275. A.D/ON237293/Argentina/2016
276. A.D/ON237298/Argentina/2017
277. A.D/ON729319/USA/2019

List of One hundred forty-five (145) complete reference genomes of RSV-A subgroup, downloaded from the RSV Genotyping Consensus Consortium (https://github.com/rsv-lineages). Data obtained on August 26, 2024.

1. B.1/MG642037/USA/1980
2. B.1/MG642044/USA/1982
3. B.1/MG642045/USA/1979
4. B.1/MG642065/USA/1981
5. B.2/MG642036/USA/1989
6. B.2/MG642054/USA/1991
7. B.2/MG642057/USA/1984
8. B.2/MG642076/USA/1987
9. B.3/MG642049/USA/1993
10. B.3/MG642064/USA/1995
11. B.4/JX198144/USA/1994
12. B.4/OK649748/USA/1992
13. B.D.1.1/KY249666/United-Kingdom/2013
14. B.D.1.1/ON237110/Argentina/2014
15. B.D.1.1/ON237115/Argentina/2014
16. B.D.1/KY249662/United-Kingdom/2013
17. B.D.1/MH594430/Kenya/2010
18. B.D.1/OK649699/Brazil/2009
19. B.D.2/MH594352/Kenya/2010
20. B.D.2/MH594457/Kenya/2010
21. B.D.2/MH594461/Kenya/2010
22. B.D.3/MH594379/Kenya/2010
23. B.D.3/MH594402/Kenya/2010
24. B.D.3/MH594407/Kenya/2010
25. B.D.3/MH594415/Kenya/2010
26. B.D.4.1.1/EPI-ISL-11055802/South-Africa/2021
27. B.D.4.1.1/EPI-ISL-11817058/Australia/2021
28. B.D.4.1.1/EPI-ISL-11817060/Australia/2021
29. B.D.4.1.1/EPI-ISL-12529656/South-Africa/2021
30. B.D.4.1.1/EPI-ISL-14769847/South-Africa/2022
31. B.D.4.1.1/EPI-ISL-15067710/Argentina/2018
32. B.D.4.1.1/EPI-ISL-15728620/South-Africa/2022
33. B.D.4.1.1/EPI-ISL-15728627/South-Africa/2022
34. B.D.4.1.1/EPI-ISL-15774019/Australia/2021
35. B.D.4.1.1/EPI-ISL-16714597/England/2021
36. B.D.4.1.1/EPI-ISL-16714599/England/2021
37. B.D.4.1.1/EPI-ISL-16714651/England/2021
38. B.D.4.1.1/MT373705/Russia/2019
39. B.D.4.1.1/MZ151850/Russia/2019
40. B.D.4.1.1/MZ515553/United-Kingdom/2019
41. B.D.4.1.1/MZ515561/United-Kingdom/2019
42. B.D.4.1.1/MZ515562/Netherlands/2018
43. B.D.4.1.1/MZ515590/United-Kingdom/2019
44. B.D.4.1.1/MZ515599/Netherlands/2019
45. B.D.4.1.1/MZ515636/United-Kingdom/2019
46. B.D.4.1.1/MZ515656/Spain/2019
47. B.D.4.1.1/MZ515658/Netherlands/2018
48. B.D.4.1.1/MZ515665/Netherlands/2017
49. B.D.4.1.1/MZ515716/United-Kingdom/2018
50. B.D.4.1.1/MZ515743/Netherlands/2017
51. B.D.4.1.1/MZ515761/Netherlands/2018
52. B.D.4.1.1/MZ515779/United-Kingdom/2018
53. B.D.4.1.1/MZ515827/Spain/2019
54. B.D.4.1.1/MZ515837/United-Kingdom/2019
55. B.D.4.1.1/MZ515904/United-Kingdom/2020
56. B.D.4.1.1/MZ515926/Netherlands/2017
57. B.D.4.1.1/MZ515975/Netherlands/2019
58. B.D.4.1.1/MZ516021/United-Kingdom/2018
59. B.D.4.1.1/MZ516054/Spain/2019
60. B.D.4.1.1/MZ516061/United-Kingdom/2018
61. B.D.4.1.1/MZ516143/Spain/2019
62. B.D.4.1.1/OM857370/Australia/2020
63. B.D.4.1.1/OM857373/Australia/2019
64. B.D.4.1.1/OM857375/Australia/2020
65. B.D.4.1.1/OM857381/Australia/2020
66. B.D.4.1.1/OM857393/Australia/2020
67. B.D.4.1.1/OP975389/Australia/2019
68. B.D.4.1.1/OQ261736/Austria/2019
69. B.D.4.1.1/OQ261743/Austria/2022
70. B.D.4.1/EPI-ISL-15120696/Morocco/2016
71. B.D.4.1/EPI-ISL-15120725/Morocco/2016
72. B.D.4.1/EPI-ISL-15120751/Morocco/2017
73. B.D.4.1/EPI-ISL-15120753/Morocco/2017

74. B.D.4.1/KY249660/United-Kingdom/2013
75. B.D.4.1/KY249683/United-Kingdom/2016
76. B.D.4.1/LC385008/Philippines/2016
77. B.D.4.1/LR699735/United-Kingdom/2017
78. B.D.4.1/LR699740/Spain/2018
79. B.D.4.1/LR699742/Spain/2017
80. B.D.4.1/MH327947/India/2017
81. B.D.4.1/MK749869/Nicaragua/2016
82. B.D.4.1/MK749877/Nicaragua/2015
83. B.D.4.1/MK749915/Nicaragua/2016
84. B.D.4.1/MT040088/USA/2016
85. B.D.4.1/MZ515674/Netherlands/2018
86. B.D.4.1/MZ516082/Netherlands/2017
87. B.D.4.1/MZ516104/Netherlands/2018
88. B.D.4.1/ON237091/Argentina/2014
89. B.D.4.1/ON237138/Argentina/2015
90. B.D.4.1/ON237168/Argentina/2015
91. B.D.4.1/ON237186/Argentina/2016
92. B.D.4.1/ON237208/Argentina/2016
93. B.D.4.1/ON237214/Argentina/2017
94. B.D.4/KY249656/United-Kingdom/2014
95. B.D.4/KY249677/United-Kingdom/2012
96. B.D.4/LC385007/Philippines/2015
97. B.D.4/MK109786/Jordan/2012
98. B.D.4/MZ516025/Netherlands/2017
99. B.D.4/OK649704/Brazil/2009
100. B.D.4/OK649726/Brazil/2010
101. B.D.4/ON237114/Argentina/2014
102. B.D.4/ON237134/Argentina/2015
103. B.D.4/ON237159/Argentina/2015
104. B.D.4/ON237167/Argentina/2015
105. B.D.E.1/OP890343/USA/2022
106. B.D.E.1/OP890347/USA/2022
107. B.D.E.1/OP965698/USA/2021
108. B.D.E.1/OP965703/USA/2021
109. B.D.E.1/OQ357797/USA/2022
110. B.D.E.2/MZ515997/Netherlands/2018
111. B.D.E.2/MZ516102/United-Kingdom/2019
112. B.D.E.2/MZ516135/Netherlands/2018
113. B.D.E.2/OM857390/Australia/2020
114. B.D.E.3/EPI-ISL-16533866/Austria/2022
115. B.D.E.3/EPI-ISL-16714385/England/2021
116. B.D.E.3/EPI-ISL-16714627/England/2021
117. B.D.E.4/EPI-ISL-15896190/Australia/2022
118. B.D.E.4/EPI-ISL-16714784/Australia/2022
119. B.D.E.4/EPI-ISL-16959502/New-Zealand/2021
120. B.D.E.4/EPI-ISL-16959704/New-Zealand/2021
121. B.D.E.4/EPI-ISL-16960135/New-Zealand/2021
122. B.D.E.4/EPI-ISL-17066801/Australia/2022
123. B.D.E.4/OM857382/Australia/2021
124. B.D.E.4/OM857387/Australia/2021
125. B.D.E.4/OM857388/Australia/2020
126. B.D/EPI-ISL-6494896/England/2008
127. B.D/KY249663/United-Kingdom/2013
128. B.D/MH594400/Kenya/2010
129. B.D/MH594451/Kenya/2010
130. B.D/MK109765/Jordan/2011
131. B.D/MK109766/Jordan/2013
132. B.D/MK109779/Jordan/2010
133. B.D/MK109780/Jordan/2013
134. B.D/MK109781/Jordan/2011
135. B.D/MW582529/Germany/2015
136. B.D/OK649702/Brazil/2009
137. B.D/OK649703/Brazil/2009
138. B.D/OK649706/Brazil/2009
139. B.D/OK649719/Brazil/2009
140. B.D/OK649720/Brazil/2009
141. B.D/OK649724/Brazil/2010
142. B.D/OK649725/Brazil/2010
143. B.D/ON237105/Argentina/2014
144. B.D/ON237140/Argentina/2015
145. B.D/ON237165/Argentina/2015

Table I: RSV genomes obtained, distributed by epidemic outbreak (2018 to 2024), antigenic subgroup, and demographic characteristics of patients in Panama

Characteristics	RSV - Epidemic Outbreaks																		Total (%)
	2018			2019-20			2021			2022			2023			2024			
	Type		Total	Type		Total	Type		Total	Type		Total	Type		Total	Type		Total	
	A	B		A	B		A	B		A	B		A	B		A	B		
No. complete genomes	8	10	18	20	9	29	0	8	8	19	1	20	10	10	20	17	3	20	115 (100)
Gender																			
Male	5	5	10	7	1	8	0	6	6	13	0	13	5	7	12	13	2	15	64 (55.7)
Female	3	5	8	13	8	21	0	2	2	6	1	7	5	3	8	4	1	5	51 (44.3)
Clinical Status																			
Hospitalized	8	10	18	19	7	26	0	3	3	0	0	0	7	5	12	16	3	19	78 (67.8)
Outpatient	0	0	0	1	1	2	0	5	5	19	1	20	3	5	8	1	0	1	36 (31.3)
Deceased	0	0	0	0	1	1	0	0	0	0	0	0	0	0	0	0	0	0	1 (0.9)
Patient age, y																			
< 1	7	9	16	15	8	23	0	4	4	6	1	7	5	4	9	11	2	13	72 (62.6)
1 - 4	1	1	2	5	1	6	0	4	4	9	0	9	2	5	7	6	1	7	35 (30.4)
5 - 50	0	0	0	0	0	0	0	0	0	4	0	4	3	1	4	0	0	0	8 (7.0)
Geographic Area																			
MA&P	4	3	7	8	1	9	0	6	6	19	1	20	8	3	11	12	3	15	68 (59.1)
The Rest of the Country	4	7	11	12	8	20	0	2	2	0	0	0	2	7	9	5	0	5	47 (40.9)

RSV, respiratory syncytial virus; "A", RSV subgroup A; "B", RSV subgroup B; MA&P, Metropolitan area and periphery.
Between 2018 and 2024, 115 complete RSV genomes were obtained, with 64.3% corresponding to RSV-A and 35.7% to RSV-B. Most genomes were from children under 1 year old (62.6%) and hospitalized patients (67.8%). Additionally, 59.1% of the cases were recorded in the metropolitan area and its periphery.

Table II: Clinical and demographic characteristics of RSV-positive cases (2018-2024) sequenced by WGS and grouped by epidemic outbreak and lineage found.

Characteristics	2018						2019-20						2021			2022						2023						
	A.D	A.D.1	A.D.2.2	A.D.3	B.D.4.1.1	Total	A.D	A.D.1	A.D.2.2.1	A.D.5	B.D.4.1.1	Total	B.D.4.1.1	B.D.E.1	Total	A.D.1	A.D.1.7	A.D.3.2	A.D.3.3	B.D.E.1	Total	A.D.1	A.D.1.5	A.D.1.7	A.D.3	A.D.5.2	B.D.4.1.1	B.D.[illegible]
No. complete genomes	3	1	2	2	10	18	2	16	1	1	9	29	1	7	8	13	2	2	2	1	20	1	1	1	2	5	6	4
Gender																												
Male	2	1	0	2	5	10	1	6	0	0	1	8	1	5	6	8	2	1	2	0	13	1	0	0	2	2	5	2
Female	1	0	2	0	5	8	1	10	1	1	8	21	0	2	2	5	0	1	0	1	7	0	1	1	0	3	1	2
Clinical Status																												
Hospitalized	3	1	2	2	10	18	2	16	0	1	7	26	1	2	3	0	0	0	0	0	0	1	1	1	1	3	4	1
Outpatient	0	0	0	0	0	0	0	0	1	0	1	2	0	5	5	13	2	2	2	1	20	0	0	0	1	2	2	3
Deceased	0	0	0	0	0	0	0	0	0	0	1	1	0	0	0	0	0	0	0	0	0	0	0	0	0	0	0	0
Patient age, y																												
< 1	3	1	2	1	9	16	1	14	0	0	8	23	1	3	4	4	2	0	0	1	7	1	1	1	1	1	2	2
1 - 4	0	0	0	1	1	2	1	2	1	1	1	6	0	4	4	6	0	1	2	0	9	0	0	0	0	2	4	1
5 - 50	0	0	0	0	0	0	0	0	0	0	0	0	0	0	0	3	0	1	0	0	4	0	0	0	1	2	0	1
Geographic Area																												
MA&P	2	0	2	0	3	7	2	5	1	0	1	9	1	5	6	13	2	2	2	1	20	0	0	1	2	5	0	3
The Rest of the Country	1	1	0	2	7	11	0	11	0	1	8	20	0	2	2	0	0	0	0	0	0	1	1	0	0	0	6	1

RSV, respiratory syncytial virus; “A”, RSV subtype A; “B”, RSV subtype B; MA&P, Metropolitan area and periphery.

Statistical association between the RSV lineages found and Demographic characteristics

Table III: Statistical Association Between RSV Lineages and Demographic Characteristics Using Chi-Square Test of Independence

Characteristics	Lineages					
	RSV-A			RSV-B		
	Chi Square	Degrees of Freedom	P-value	Chi Square	Degrees of Freedom	P-value
Gender	12.2	10	0.272	0.730	1	0.393
Clinical Status	16.32	10	0.091	10.98	2	0.004
Patient age, y	13.29	10	0.208	3.439	2	0.179
Geographic Area	19.6	10	0.033	14.470	1	0.0001

The Chi Square and p-values obtained from the analysis are shown by lineages, separated by subgroups.
For clinical status, in RSV-A lineages the deceased variable was not considered due to insufficient data.
For patient age, in RSV-A lineages the 5-50 age range was not considered due to insufficient data.

Analysis performed at https://www.openepi.com/RbyC/RbyC.htm

Table IV: RSV lineages involved in the 2018-2024 epidemic outbreaks based on the clinical and demographic characteristics of the patients

Characteristics	RSV-A Lineages											Total RSV-A	RSV-B Lineages		Total RSV-B	Total (%)
	A.D.	A.D.1	A.D.1.5	A.D.1.7	A.D.2.2	A.D.2.2.1	A.D.3	A.D.3.2	A.D.3.3	A.D.5	A.D.5.2		B.D.4.1.1	B.D.E.1		
No. complete genomes	5	31	1	3	2	1	7	8	2	1	13	74	26	15	41	115 (100)
Gender																
Male	3	16	0	2	0	0	6	6	2	0	8	43	12	9	21	64 (55.7)
Female	2	15	1	1	2	1	1	2	0	1	5	31	14	6	20	51 (44.3)
Clinical Status																
Hospitalized	5	18	1	1	2	0	6	5	0	1	11	50	22	6	28	78 (67.8)
Outpatient	0	13	0	2	0	1	1	3	2	0	2	24	3	9	12	36 (31.3)
Deceased	0	0	0	0	0	0	0	0	0	0	0	0	1	0	1	1 (0.9)
Patient age, y																
< 1	4	20	1	3	2	0	4	3	0	0	7	44	20	8	28	72 (62.6)
1 - 4	1	8	0	0	0	1	2	4	2	1	4	23	6	6	12	35 (30.4)
5 - 50	0	3	0	0	0	0	1	1	0	0	2	7	0	1	1	8 (7.0)
Geographic Area																
MA&P	4	18	0	3	2	1	5	3	2	0	13	51	5	12	17	68 (59.1)
The Rest of the Country	1	13	1	0	0	0	2	5	0	1	0	23	21	3	24	47 (40.9)

RSV, respiratory syncytial virus; “A”, RSV subtype A; “B”, RSV subtype B; MA&P, Metropolitan area and periphery.

Table V: Information about the RSV strains isolated and those selected for the neutralization assay with mAbs

Subgroup	Strain Name	Ct values (original sample)	Lineage	F Protein Mutations (compared against NCBI references)				F Protein Mutations (GISAID references)	NT
				Binding Site			*Other mutations*		
				Nirsevimab	Motavizumab	Clersovimab			
RSV-A									
**1-**	**hRSV/A/Panama/ICGES-215/2018**	**23.2**	**A.D**	**-**	**N276S**	**M447V**	**L4P, A8T, T16A, F20L, G25N, P102A, T103A, N105S, L119I, A122T, K124N, V139G, V152I, L178V, I379V, V384I, A518V, S540A**	**L119I, A518V**	**yes**
2-	hRSV/A/Panama/ICGES-198/2018	19.7	A.D.3	-	N276S	M447V	L4P, A8T, T12I, T16A, F20L, G25N, P102A, T103A, N105S, A122T, K124N, V139G, V152I, L178V, I379V, V384I, S540A	T12I, S25N	no
3-	hRSV/A/Panama/ICGES-97/2019	21.1	A.D.1	-	N276S	M447V	L4P, A8T, T16A, F20L, G25S, P102A, T103A, N105S, A122T, K124N, V139G, V152I, L178V, I379V, V384I, S540A	-	no
4-	hRSV/A/Panama/ICGES-127/2019	20.0	A.D.1	-	N276S	M447V	L4P, A8T, T16A, F20L, G25S, P102A, T103A, N105S, A122T, K124N, V139G, V152I, L178V, I379V, V384I, S540A	-	no
5-	hRSV/A/Panama/ICGES-131/2019	20.6	A.D.1	-	N276S	M447V	L4P, A8T, T16A, F20L, G25S, P102A, T103A, N105S, A122T, K124N, V139G, V152I, N165S, L178V, I379V, V384I, S540A	N165S	no
6-	hRSV/A/Panama/ICGES-134/2019	20.4	A.D.1	-	N276S	M447V	L4P, A8T, T16A, F20L, G25S, P102A, T103A, N105S, A122T, K124N, V139G, V152I, L178V, I379V, V384I, S540A	-	no
7-	hRSV/A/Panama/ICGES-28/2022	22.1	A.D.1	-	N276S	M447V	L4P, A8T, T13A, T16A, F20L, G25S, P102A, T103A, N105S, A122T, K124T, V139G, V152I, L178V, I379V, V384I, S540A	T13A, N124T	no

Table V: Information about the RSV strains isolated and those selected for the neutralization assay with mAbs

Subgroup	Strain Name	Ct values (original sample)	Lineage	F Protein Mutations (compared against NCBI references)				F Protein Mutations (GISAID references)	NT
				Binding Site			*Other mutations*		
				Nirsevimab	Motavizumab	Clersovimab			
RSV-A									
8-	hRSV/A/Panama/ICGES-32/2022	22.8	A.D.1	-	N276S	M447V	L4P, A8T, T13A, T16A, F20L, G25S, P102A, T103A, N105S, A122T, K124T, V139G, V152I, L178V, I379V, V384I, S540A	T13A, N124T	no
9-	hRSV/A/Panama/ICGES-37/2022	24.8	A.D.1	-	N276S	M447V	L4P, A8T, T13A, T16A, F20L, G25S, P102A, T103A, N105S, A122T, K124T, V139G, V152I, L178V, I379V, V384I, S540A	T13A, N124T	no
10-	hRSV/A/Panama/ICGES-40/2022	18.8	A.D.1	-	N276S	M447V	L4P, A8T, T13A, T16A, F20L, G25S, P102A, T103A, N105S, A122T, K124T, V139G, V152I, L178V, I379V, V384I, S540A	T13A, N124T	no
**11-**	**hRSV/A/Panama/ICGES-45/2022**	**20.7**	**A.D.1**	-	**N276S**	**M447V**	**L4P, A8T, T13A, T16A, F20L, G25S, P102A, T103A, N105S, A122T, K124T, V139G, V152I, L178V, I379V, V384I, S540A**	**T13A, N124T**	**yes**
12-	hRSV/A/Panama/ICGES-43/2022	21.0	A.D.1	-	N276S	M447V	L4P, A8T, T13A, T16A, F20L, G25S, P102A, T103A, N105S, A122T, K124T, V139G, V152I, L178V, I379V, V384I, S540A	T13A, N124T	no
13-	hRSV/A/Panama/ICGES-44/2022	19.8	A.D.1	-	N276S	M447V	L4P, A8T, T13A, T16A, F20L, G25S, P102A, T103A, N105S, A122T, K124T, V139G, V152I, L178V, I379V, V384I, S540A	T13A, N124T	no
14-	hRSV/A/Panama/ICGES-59/2022	23.1	A.D.1	-	N276S	M447V	L4P, A8T, T13A, T16A, F20L, G25S, P102A, T103A, N105S, A122T, K124T, V139G, V152I, L178V, I379V, V384I, S540A	T13A, N124T	no
15-	hRSV/A/Panama/ICGES-60/2022	21.6	A.D.1	-	N276S	M447V	L4P, A8T, T13A, T16A, F20L, G25S, P102A, T103A, N105S, A122T, K124T, V139G, V152I, L178V, I379V, V384I, S540A, K551R	T13A, N124T, K551R	no

Table V: Information about the RSV strains isolated and those selected for the neutralization assay with mAbs

Subgroup	Strain Name	Ct values (original sample)	Lineage	F Protein Mutations (compared against NCBI references)				F Protein Mutations (GISAID references)	NT
				Binding Site			*Other mutations*		
				Nirsevimab	Motavizumab	Clersovimab			
RSV-A									
**16-**	**hRSV/A/Panama/ICGES-42/2022**	**22.0**	**A.D.3.2**	**-**	**-**	**M447V**	**L4P, A8T, T12I, T16A, F20L, G25S, P102A, T103A, N105S, A122T, K124N, V139G, V152I, L178V, I379V, V384I, S540A**	**T12I, S276N**	**yes**
**17-**	**hRSV/A/Panama/ICGES-147/2023**	**18.0**	**A.D.3**	**-**	**N276S**	**M447V**	**L4P, A8T, T12I, T16A, F20L, G25S, P102A, T103A, N105S, M115I, A122T, K124N, V139G, V152I, L178V, I379V, V384I, S540A**	**T12I, M115I**	**yes**
18-	hRSV/A/Panama/ICGES-343/2023	16.8	A.D.3	-	N276S	M447V	L4P, A8T, T12I, T16A, F20L, G25S, P102A, T103A, N105S, M115I, A122T, K124N, V139G, V152I, L178V, I379V, V384I, S540A	T12I, M115I	no
RSV-B									
**1-**	**hRSV/B/Panama/ICGES-114/2019**	**20.8**	**B.D.4.1.1**	***I206M and Q209R***	**-**	**-**	**L8S, F45L, A103V, L172Q, S173L, K191R, N234T, T529A,**	**-**	**yes**

"RSV", respiratory syncytial virus; "RSV-A", RSV subgroup A; "RSV-B", RSV subgroup B; "NT", Neutralization Test.
For the analysis of mutations in the F protein, reference sequences from NCBI GenBank (NC_038235 for RSV-A and NC_001781 for RSV-B) and EpiRSV GISAID (EPI_ISL_412866 for RSV-A and EPI_ISL_1653999 for RSV-B) were used.
Nirsevimab binding site in RSV F protein: Residues 62–69 in the F2 subunit and 196–212 in the F1 subunit (Zhu et al., 2017).
Motavizumab binding site in RSV F protein: Residues 255–277 in the F1 subunit (Mas et al., 2018).
Clersovimab binding site in RSV F protein: Residues E161, S182, and 426–447 in the F1 subunit (Tang et al., 2019).

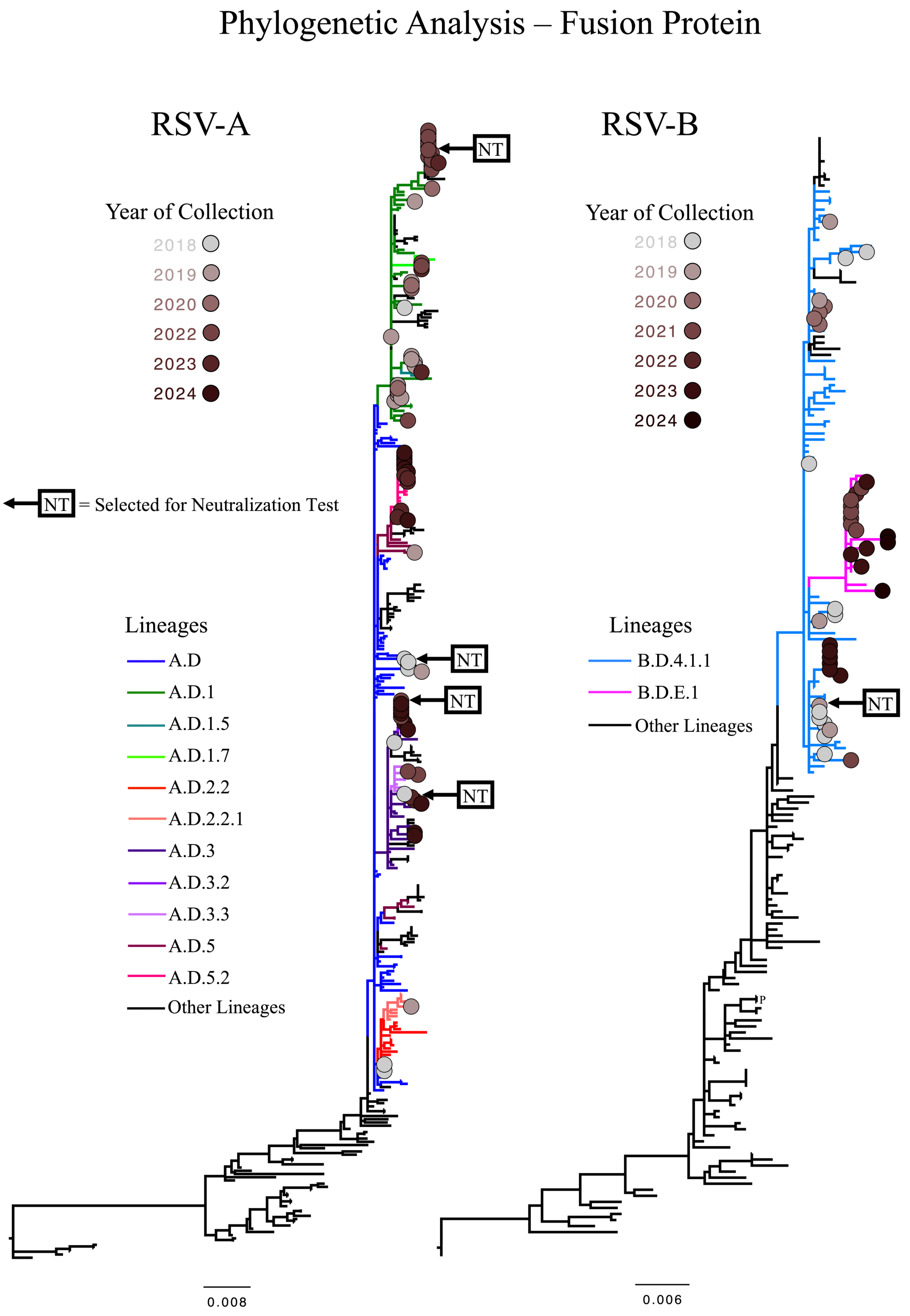

Figure I: Maximum-likelihood phylogenetic trees of *Fusion gen* of RSV-A and RSV-B grouped with reference strains. RSV genomes from Panama are highlighted with circles ranging from light red (2018) to dark red (2024) at branch tips. RSV subgroups are represented by the colors of the tree branches. The isolated strains subjected to neutralization assays (NT) against the F protein are indicated in the tree. The scale bar indicates nucleotide substitutions per site. "RSV-A", RSV subgroup A; "RSV-B", RSV subgroup B, "NT", Neutralization Test.

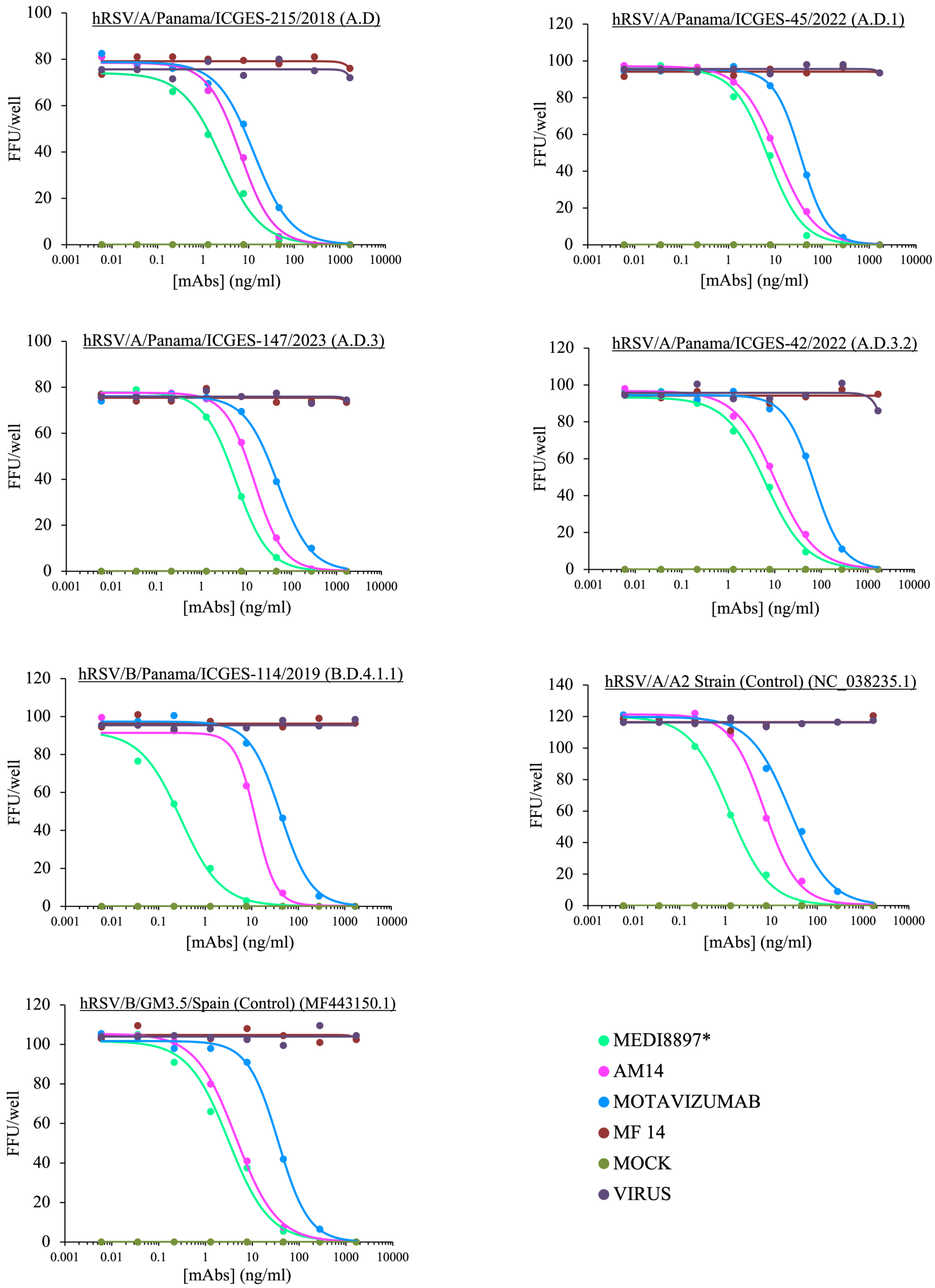

Figure II: Determination of IC50 values for RSV isolates. Each graph corresponds to a specific strain, indicated at the top of each plot. Neutralization assays were performed using monoclonal antibodies (mAbs), with their respective concentrations (ng/mL) shown on the x-axis. The y-axis represents the number of FFU counted in each well. mAbs are color-coded as follows: MEDI8897* (turquoise), AM14 (magenta), MOTAVIZUMAB (blue) and MF 14 (green). IC50 values were calculated by fitting the experimental data to a four-parameter logistic (4PL) equation using Microsoft Excel (Sebaugh, 2011). "hRSV", human Respiratory Syncytial Virus; "FFU", Focus Forming Units; "mAbs", Monoclonal Antibodies; "ng", Nanograms; "ml", Milliliters.

Statistical association analysis between the antigenic subgroups found and the gender of the patients

Fisher's Exact Test (p-value 2-tail)

Contingency tables (data) for Gender: (Male vs Female) for RSV Subgroups A vs B for each epidemic outbreak (2018-2024)

2018: [[6, 6], [4, 9]], Male vs Female, P-value: 0.5682

2019-20: [[31, 9], [25, 11]], Male vs Female, P-value: 0.5920

2021: [[0, 10], [0, 5]], Male vs Female, P-value: >0.9999

2022: [[34, 2], [26, 3]], Male vs Female, P-value: 0.7932

2023: [[19, 24], [12, 8]], Male vs Female, P-value: 0.3694

2024: [[30, 5], [21, 2]], Male vs Female, P-value: 0.8394

2018-2024: [[120, 56], [88, 38]], Male vs Female, P-value: 0.9840

There is no statistically significant association between the variables

Analysis performed at https://www.openepi.com/TwobyTwo/TwobyTwo.htm

Statistical association analysis between the antigenic subgroups found and the clinical status of the patients

Fisher's Exact Test (p-value 2-tail)

Contingency tables (data) for Clinical Status: (Hospitalized vs Outpatient) for RSV Subgroups A vs B for each epidemic outbreak (2018-2024)

2018: [[10, 15], [0, 0]], Hospitalized vs Outpatient, P-value: >0.9999

2019-20: [[53, 17], [3, 2]], Hospitalized vs Outpatient, P-value: 0.7487

2021: [[0, 4], [0, 11]], Hospitalized vs Outpatient, P-value: >0.9999

2022: [[7, 2], [53, 3]], Hospitalized vs Outpatient, P-value: 0.2747

2023: [[21, 18], [9, 13]], Hospitalized vs Outpatient, P-value: 0.4822

2024: [[48, 7], [3, 0]], Hospitalized vs Outpatient, P-value: >0.9999

2018-2024: [[139, 63], [68, 29]], Hospitalized vs Outpatient P-value: 0.9310

There is no statistically significant association between the variables

Analysis performed at https://www.openepi.com/TwobyTwo/TwobyTwo.htm

Statistical association between the antigenic subgroups found and the Patient age of the patients

Fisher's Exact Test (p-value 2-tail)

Contingency tables (data) for Patient age, y: (<1 vs 1-4) for RSV Subgroups A vs B for each epidemic outbreak (2018-2024)

2018: [[9, 13], [1, 2]], Age group < 1 vs 1-4, P-value: >0.9999

2019-20: [[38, 16], [17, 4]], 2019-20 Age group < 1 vs 1-4, P-value: 0.5316

2021: [[0, 4], [0, 8]], Age group < 1 vs 1-4, P-value: >0.9999

2022: [[28, 2], [24, 3]], Age group < 1 vs 1-4, P-value: 0.8978

2023: [[14, 15], [9, 14]], Age group < 1 vs 1-4, P-value: 0.7065

2024: [[27, 3], [22, 4]], Age group < 1 vs 1-4, P-value: 0.8354

2018-2024: [[116, 53], [73, 35]], Age group < 1 vs 1-4, 0.9572

There is no statistically significant association between the variables

Analysis performed at https://www.openepi.com/TwobyTwo/TwobyTwo.htm

Statistical association between the antigenic subgroups found and the Geographic Area of the patients

Fisher's Exact Test (p-value 2-tail)

Contingency tables (data) for Geographic Area: (MA&P vs The Rest of the Country) for RSV Subgroups A vs B for each epidemic outbreak (2018-2024)

2018: [[5, 5], [5, 10]], MA&P vs The Rest of the Country, P-value: 0.6748

2019-20: [[17, 6], [39, 14]], MA&P vs The Rest of the Country, P-value: >0.9999

2021: [[0, 9], [0, 6]], MA&P vs The Rest of the Country, P-value: >0.9999

2022: [[50, 4], [10, 1]], MA&P vs The Rest of the Country, P-value: >0.9999

2023: [[21, 10], [10, 23]], MA&P vs The Rest of the Country, P-value: 0.0056

2024: [[38, 6], [13, 1]], 2024 MA&P vs The Rest of the Country, P-value: 0.9123

2018-2024: [[131, 40], [77, 55]], 2024 MA&P vs The Rest of the Country, P-value: 0.001074

Analysis performed at https://www.openepi.com/TwobyTwo/TwobyTwo.htm

Statistical association between the RSV-B lineages found and the Clinical Status of the patients

Fisher's Exact Test (p-value 2-tail)

Contingency tables (data) for Clinical Status: (Hospitalized vs Outpatient) for RSV Lineages B.D.4.1.1 vs B.D.E.1 for each epidemic outbreak (2018-2024)

2018-2024: [[22, 6], [3, 9]], Hospitalized vs Outpatient, P-value: 0.004456

Analysis performed at https://www.openepi.com/TwobyTwo/TwobyTwo.htm

Statistical association between the RSV-B lineages found and the Geographic Area of the patients

Fisher's Exact Test (p-value 2-tail)

Contingency tables (data) for Geographic Area: (MA&P vs The Rest of the Country) for RSV Lineages B.D.4.1.1 vs B.D.E.1 for each epidemic outbreak (2018-2024)

2018-2024: [[5, 12], [21, 3]], MA&P vs The Rest of the Country, P-value: 0.0005

Analysis performed at https://www.openepi.com/TwobyTwo/TwobyTwo.htm
